# Machine learning-based algorithms applied to drug prescriptions and other healthcare services in the Sicilian claims database to identify acromegaly as a model for the earlier diagnosis of rare diseases

**DOI:** 10.1038/s41598-024-56240-w

**Published:** 2024-03-14

**Authors:** Salvatore Crisafulli, Andrea Fontana, Luca L’Abbate, Giacomo Vitturi, Alessia Cozzolino, Daniele Gianfrilli, Maria Cristina De Martino, Beatrice Amico, Carlo Combi, Gianluca Trifirò

**Affiliations:** 1 Department of Medicine, University of Verona, Verona, Italy; 2 Unit of Biostatistics, Fondazione IRCCS Casa Sollievo Della Sofferenza, San Giovanni Rotondo, Italy; 3 Department of Biomedical and Dental Sciences and Morphofunctional Imaging, University of Messina, Messina, Italy; 4 Department of Diagnostics and Public Health, University of Verona, P.Le L.A. Scuro 10, 37124 Verona, Italy; 5 Section of Medical Pathophysiology and Endocrinology, Department of Experimental Medicine, Sapienza University of Rome, Rome, Italy; 6 Dipartimento Di Medicina Clinica E Chirurgia, Università Federico II Di Napoli, Naples, Italy; 7 Department of Computer Science, University of Verona, Verona, Italy

**Keywords:** Endocrinology, Epidemiology

## Abstract

Acromegaly is a rare disease characterized by a diagnostic delay ranging from 5 to 10 years from the symptoms’ onset. The aim of this study was to develop and internally validate machine-learning algorithms to identify a combination of variables for the early diagnosis of acromegaly. This retrospective population-based study was conducted between 2011 and 2018 using data from the claims databases of Sicily Region, in Southern Italy. To identify combinations of potential predictors of acromegaly diagnosis, conditional and unconditional penalized multivariable logistic regression models and three machine learning algorithms (i.e., the Recursive Partitioning and Regression Tree, the Random Forest and the Support Vector Machine) were used, and their performance was evaluated. The random forest (RF) algorithm achieved the highest Area under the ROC Curve value of 0.83 (95% CI 0.79–0.87). The sensitivity in the test set, computed at the optimal threshold of predicted probabilities, ranged from 28% for the unconditional logistic regression model to 69% for the RF. Overall, the only diagnosis predictor selected by all five models and algorithms was the number of immunosuppressants-related pharmacy claims. The other predictors selected by at least two models were eventually combined in an unconditional logistic regression to develop a meta-score that achieved an acceptable discrimination accuracy (AUC = 0.71, 95% CI 0.66–0.75). Findings of this study showed that data-driven machine learning algorithms may play a role in supporting the early diagnosis of rare diseases such as acromegaly.

## Introduction

Acromegaly is a chronic and progressive endocrine rare disease characterized by an overproduction of growth-hormone (GH) and elevated insulin-like growth factor 1 (IGF-1) levels, typically resulting from a GH-secreting pituitary adenoma^1^. Acromegaly globally affects around 6 per 100,000 persons, with an incidence of 3.8 cases per million per year^2^. Prevalence of acromegaly in Italy ranges from 6.9 to 9.7 cases per 100,000 persons^3–5^. Clinical manifestations of acromegaly mainly include morphological changes, cardiovascular disorders, osteoarticular and metabolic manifestations, sleep apnea and respiratory diseases^6,7^. The recommended diagnostic test for acromegaly consists of serum IGF-1 levels measurement and, in case of elevated or equivocal IGF-1, the diagnosis must be confirmed with lack of suppression of GH to < 1 μg/L, following documented hyperglycemia during an oral glucose load. Biochemical diagnosis should be thereafter confirmed by radiological evaluation, with magnetic resonance imaging (MRI) being the gold standard, replaced by computed tomography (CT) scan if MRI is contraindicated or unavailable^8^.

A survey conducted in 2013 reported that rare patients need, on average, more than 5 years to receive a correct diagnosis, usually after having received around three misdiagnoses and inappropriate treatments^9^. Late diagnosis in rare diseases is often due to insufficient knowledge and lack of awareness of patients/clinicians or to the heterogeneity of rare disease manifestations^10^, thus increasing the difficulty for the clinicians to make a correct diagnosis. Concerning acromegaly, despite the improvement in diagnostic techniques over the years, it is often diagnosed 5—10 years after onset symptoms^11,12^, mainly due to its slow onset and its non-specific signs and symptoms which lead patients to refer to different medical professionals who may fail to diagnose acromegaly^13^. The delay in diagnosis may have an extremely negative impact on different social and health aspects, including long-term disease prognosis, the treatment success rate, psychosocial impairment^14,15^. (e.g., depression, daytime sleepiness, sleep disturbances, disturbances of body image, and quality of life) and mortality^6,11,12,16^. Therefore, reducing the diagnostic delay and anticipating the surgical/pharmacological treatment of the disease is a crucial point in the management of acromegalic patients.

Especially in the field of rare diseases, artificial intelligence (AI) and machine learning techniques, which are increasingly applied in medicine and healthcare^17^, might help physicians to earlier identify rare diseases and timely refer patients to specialist centers. Indeed, computers can play a key role by collecting and learning considerable quantities of digital information, especially concerning prescriptions of drugs and diagnostic tests. This large amount of information collected during daily routine care can be useful features for a machine learning model to find a statistical pattern that can help physicians identifying conditions that they usually do not encounter frequently in practice^17,18^.

The aim of the study was to develop, internally validate, and compare different machine-learning algorithms to identify a combination of drug prescriptions and other healthcare services for the early diagnosis of acromegaly in a Southern Italian population using administrative claims databases.

## Methods

### Data source

This Italian, retrospective, population-based study was conducted between January 2011 and December 2018 using data from the fully anonymized claims databases of Sicily Region, with an average of 5,031,655 inhabitants. This database contains demographic and medical data that is collected through services provided by the Italian National Health Service (NHS). It includes information on demographics of residents in Sicily Region, outpatient pharmacy claims, hospital discharges, exemptions from co-payment, referrals for outpatient diagnostic tests and specialist’s visits database. The dispensed drugs were coded using the Anatomical Therapeutic Clinical (ATC) classification system and the Italian Marketing Authorization Code (AIC), while comorbidities were coded through the ninth revision of the International Classification of Diseases—Clinical Modification (ICD-9-CM).

### Acromegaly cohort definition

Using a validated coding algorithm^19^, acromegaly cases were identified as those subjects who had claims suggestive of acromegaly in at least two of the following data sources: (i) hospital discharge records (ICD-9-CM code: 253.0); (ii) exemption from co-payment (exemption codes: 001, 253.0); (iii) pharmacy claims for somatostatin analogues (i.e., octreotide, ATC: H01CB02; lanreotide, ATC: H01CB03; pasireotide, ATC: H01CB05) and/or pegvisomant (ATC: H01AX01); (iv) prescriptions for facial bone nuclear magnetic resonance (88.91.3–88.91.4) and/or cranial CT (87.03–87.03.1) and/or somatotropic hormone measurement (88.97, 90.35.1) and/or IGF-1 levels measurement (90.40.6), during the specialist examinations.

For each identified acromegaly case, the date of the first claim for at least one of the above-mentioned conditions was considered as the index date (ID).

### Definition and selection of controls (matching criteria)

Cases were matched with up to 10 controls (not affected by acromegaly) extracted from the same data source by date of birth (± 2 years), gender, and database history using the exact method (i.e., matching each case to all possible controls with the same values on the two above mentioned matching features). Database history indicates the timeframe elapsing between the first claim of the patient in the database and his/her index date. Controls were selected from a random sample of almost 180,000 subjects registered in Sicilian claims databases. For each paired control, the same ID of the corresponding matched case was assigned. All controls who deceased prior to the ID of the corresponding matched case and all controls with no claims in any of the data sources before the ID of the corresponding matched case were excluded from the matching set. The matching procedure was performed by using a user-defined macro written in standard SAS language (SAS Software, Release 9.4, SAS Institute, Cary, NC, USA). The SAS code is available upon request.

### Features list

To predict acromegaly diagnosis, the following features (i.e., predictors) were considered: (1) the presence of some pre-existing comorbidities associated to the acromegaly (identified through specific coding algorithms reported in Supplementary Table 1); (2) the presence and the frequency of drug dispensing (both at II and V ATC level, separately) in the outpatient pharmacy claims database; (3) the presence and the frequency of specialist visits or laboratory/diagnostic tests from the diagnostic tests and specialist’s visits database; (4) the presence of any exemption from co-payment for each identified code separately; (5) the presence of any hospitalization for each identified diagnostic code separately. The presence of comorbidities, exemptions from co-payment and hospitalizations was assessed any time prior to ID, while drug dispensing and specialist visits or laboratory/diagnostic tests were computed at different time windows before the ID.

### Time window selection

The database history among cases and controls in the Sicilian Regional claims database, considering the different data sources separately (Supplementary Fig. 1). As more than 50% of the cases and controls had at least 2 years (i.e., the median value of the time distribution rounded to the nearest integer) of database history, especially concerning pharmacy claims and specialist visits or laboratory/diagnostic tests data sources, the time window for the main analysis was set up at 2 years. As for the hospital discharges and exemption from co-payment claims data, the database history was longest in the controls (i.e., more than 75% of them had at least one year of database history); on the other hand, about 75% (upper quartile range) of both cases and controls had about 3 and 5 years of database history in the two data sources, respectively. For this reason, timeframes of 1, 3, 4 and 5 years were also evaluated in the sensitivity analyses.

### Descriptive statistics and univariable analysis

Continuous variables were reported as mean ± standard deviation (SD), median along with interquartile range (IQR) whereas categorical variables as absolute and relative frequencies (percentages).

The association between each candidate predictor and the presence of acromegaly was assessed using over-dispersed Poisson regression or conditional logistic regression models for count and binary predictors, respectively. Conditional logistic regression is an extension of the classical (i.e., unconditional) logistic regression that allows for stratification due to matching sets. Mean ratios and odds ratios were estimated from the two models respectively, along with their 95% confidence intervals (CIs) and p-values have been corrected for multiple testing, following the Bonferroni method. Statistical significance was claimed for *p* < 0.05.

### Development and validation of machine learning predictive algorithms

To identify possible linear and non-linear combinations of candidate predictors, associated with the diagnosis of acromegaly, two different logistic regression models and three machine learning algorithms were performed: (1) Cross-validated multivariable conditional logistic model with Least Absolute Shrinkage and Selection Operator (LASSO) penalty (CLOGIT); (2) Cross-validated multivariable unconditional logistic model with LASSO penalty; (3) Recursive PArtitioning and Regression Tree (RPART); (4) Random Forest (RF), using the probabilistic version^20^; (5) Support Vector Machine (SVM), using the probabilistic version. In the probabilistic version, the algorithms assign to each subject an individual predicted probability of having the disease diagnosis.

Basically, each proposed predictive model or algorithm identifies the most strongly associated predictors (among all candidate ones) and return either a vector of estimated individual probability of having the disease (i.e., unconditional logistic regression model, RPART, probabilistic RF and probabilistic SVM) or a binary classification (i.e., CLOGIT). From now on, for the sake of simplicity, both models and algorithms were referred to as 'algorithms'.

Each algorithm was developed (i.e., built) exclusively on a random sample of the original dataset (i.e., the training set, defined by including a random selection of about 70% of the original observations and preserving the integrity of the case–control matching set) while its performance was always assessed in the remaining 30% of data not included in the training set (i.e., test set). All the algorithms were built on the same training set and their performance was evaluated on the same test set. During the training step, the problem of overfitting the algorithm to the observed data may arise. This problem was only and exclusively addressed during the algorithm training and not during its validation (testing). To minimize the overfitting of the algorithm, different actions were taken depending on the type of algorithm considered: a tenfold Cross-Validation (CV) of the training dataset was performed both for LASSO and RPART to robustly select all the features and prune trees, respectively. Also in the SVM, a tenfold CV was performed to detect the optimal cost and gamma parameters which maximize the accuracy whereas, in the RF, a sort of internal CV known as “out-of-bag” (OOB) estimation was used to assess the prediction accuracy of each tree of the forest in unseen data. In this process, each tree of the forest was built using a different bootstrap sample from the training dataset. About one-third of the observations are left out of the bootstrap sample and not used in the building of each tree. This OOB data is then used to get a running unbiased estimate of the prediction error as trees are added to the forest and to get estimates of variable importance. Furthermore, as the number of cases was extremely lower than the number of controls, in order to account for this sample size imbalance and increase the accuracy in correctly predicting the probability of detecting cases, different weights were allocated to cases and controls, only in the training dataset, when running both the tree-based (i.e., RPART and RF) and the SVM algorithms, following the inverse probability weight (IPW) method. For each algorithm, the optimal hyperparameters values were set after a “tuning phase”, choosing those that would minimize the CV error or maximize their performance following a grid search. Further details and peculiarities of the machine learning algorithms used for the early diagnosis of acromegaly are provided in Supplementary Document 1.

The performance of these algorithms was assessed in terms of discrimination (i.e., the ability of the algorithm to assign a higher probability of having the diagnosis of acromegaly in cases than in controls or, in presence of an algorithm that provides a binary classification, the ability of correctly classifying them) and in terms of calibration (i.e., the ability of the algorithm to assign predicted probabilities that are aligned with the observed frequencies). For those algorithms that return a vector of estimated individual probabilities, the discriminatory ability was assessed by the area under the Receiver Operator Characteristic (ROC) curve (AUC) on these probabilities (also referred to as the C-statistic), along with 95% CI computed using the DeLong method. A generally accepted approach suggests that an area under the ROC curve or C statistic of less than 0.70 is considered poor discrimination; between 0.70 to 0.79 is considered acceptable discrimination; between 0.80 to 0.89 is considered excellent discrimination and more than or equal to 0.90 is considered outstanding discrimination^21^. The optimal threshold on predicted probabilities was detected in the ROC curve space as the one which maximizes the Youden index. The optimal threshold was also used to provide a binary classification for clinical purposes only (e.g., above the cut-off the subject would be classified as a case and below the cut-off as a control) and the following diagnostic measures were reported: sensitivity, specificity, positive predictive value (PPV), negative predictive value (NPV) and F-score. Moreover, the goodness of fit of the predicted probabilities (i.e., calibration) was assessed by the integrated calibration index (ICI)^22^ and is often considered a non-negligible feature of the algorithm (i.e., poorly calibrated algorithms will underestimate or overestimate the outcome of interest).

A comparative analysis of the performance of all algorithms was carried out and the best algorithm was defined as the one with the highest AUC (or Youden index where appropriate) in the test set and simultaneously using the fewest predictors (the most parsimonious). Finally, all predictors identified by at least two different algorithms were included in an unconditional multivariable logistic regression model to build a “meta-score” for the prediction of acromegaly diagnosis. This study was conducted and reported according to the Transparent Reporting of a multivariate prediction model for Individual Prediction or Diagnosis (TRIPOD) guidelines^23^. All statistical analyses were carried out using the R Foundation for Statistical Computing software (ver. 4.0, packages: “clogitL1”, "glmnet","party", "ranger", “rpart”, “pROC”, “caret”, “rminer”).

### Assessing the diagnostic accuracy of machine-learning algorithms in absence of a gold standard test

As stated above, as acromegaly cases were identified through the use of a validated coding algorithm^19^, rather than a well-established gold standard test, they are subject to misclassification. As a result, measures of the diagnostic ability of machine-learning algorithms may be biased or inaccurate. The aim of this analysis was to quantify the bias in these estimates by comparing them with those that would have been obtained if the machine-learning algorithm had been evaluated against the gold standard test. Knowing the diagnostic ability (e.g., sensitivity and specificity) of the coding algorithm (i.e., that now acts as a “reference standard” test) and the diagnostic ability of a machine-learning algorithm against the reference standard test, it is possible to retrieve the sensitivity and specificity of the machine-learning algorithm by following the method proposed by Habibzadeh^24^. The bias was defined as the absolute difference between the sensitivity (or specificity) observed and that which would have been found if the gold standard had been used and was also estimated with respect to specific combinations of sensitivity and specificity (i.e., from 0 to 100% by 25%) detectable in a ROC curve. Further details are provided in Supplementary Document 2.

### Ethical approval

Analyses were conducted in accordance with the ethical standards of the institutional and national research committee and with the 1964 Helsinki Declaration and its later amendments. This study was approved by the Ethics Committee of the *Azienda Ospedaliera Universitaria Integrata* of Verona, Italy (Protocol number 55986, 27th September 2021). Informed consent was not necessary as there was not direct interaction with subjects, as stated by the Italian Medicines Agency in “*Determinazione AIFA 20 marzo 2008*—*Linee guida per la classificazione e conduzione degli studi osservazionali sui farmaci*”.

## Results

The target population identified in Sicilian Regional claims databases during the study period consisted of 533 patients. These cases were matched to 5,255 controls. The median age at ID was 55.0 (IQR 45.0—67.0) years and about 54% were females (matching factors) in both groups. Diabetes mellitus was the most prevalent comorbidity among both cases and controls (22.1% vs 15.5%, respectively), followed by osteoporosis (6.2% vs 5.2%, respectively) and cardiomyopathy (3.2 vs 0.2%, respectively). Demographics and baseline characteristics are shown in Table 1. Overall, the mean number (± SD) of pharmacy claims and specialist visits or laboratory/diagnostic tests within 2 years prior to ID was higher for cases (39.4 ± 46.1 and 51.8 ± 76.1, respectively) than for controls (25.0 ± 37.9 and 27.2 ± 34.9, respectively), as well as the number of previous hospitalizations (Table 1).

**Table 1:** Demographic and clinical characteristics of identified patients with acromegaly (cases) and matched controls.

		Cases N = 533	Controls^#^ N = 5,255
Matching factors			
Age at ID (years)	Mean ± SD	55.0 ± 15.6	55.3 ± 15.3
	Median [IQR]	55.0 [45.0–67.0]	55.0 [45.0–67.0]
	Range	4–90	2–92
Age at ID classes (years)—N (%)	< 18	10 (1.9)	102 (1.9)
	18–44	116 (21.8)	1,120 (21.3)
	45–64	253 (47.5)	2,475 (47.1)
	65–80	137 (25.7)	1,374 (26.1)
	> 80	17 (3.2)	184 (3.5)
Gender—N (%)	Male	245 (46.0)	2,398 (45.6)
	Female	288 (54.0)	2,857 (54.4)
Clinical characteristics			
Comorbidities*—N (%)	Inflammatory bowel diseases	11 (2.1)	4 (0.1)
	Colon polyp	3 (0.6)	6 (0.1)
	Colon cancer	0 (0.0)	5 (0.1)
	Rheumatoid arthritis	5 (0.9)	3 (0.1)
	Osteoarthritis	5 (0.9)	25 (0.5)
	Osteoporosis	33 (6.2)	272 (5.2)
	Arthropathy/arthralgia/synovitis	1 (0.2)	3 (0.1)
	Psoriatic arthritis	1 (0.2)	2 (0.0)
	Carpal tunnel syndrome	1 (0.2)	0 (0.0)
	Multiple sclerosis	11 (2.1)	1 (0.0)
	Prophylaxis of organ rejection	13 (2.4)	1 (0.0)
	Cardiomyopathy	17 (3.2)	8 (0.2)
	Cardiac hypertrophy	2 (0.4)	2 (0.0)
	Heart failure	6 (1.1)	49 (0.9)
	Cardiac dysrhythmia/arrhythmia	12 (2.3)	87 (1.7)
	Cerebrovascular disease	4 (0.8)	23 (0.4)
	Diabetes mellitus	118 (22.1)	817 (15.5)
	Sleep apnea	8 (1.5)	6 (0.1)
	Chronic kidney disease	10 (1.9)	60 (1.1)
	Menstrual abnormality	3 (0.6)	12 (0.2)
	Hypopituitarism	4 (0.8)	0 (0.0)
Number of pharmacy claims and specialist visits or laboratory/diagnostic tests, within two years prior the ID			
Individual pharmacy claims	Mean ± SD	39.4 ± 46.1	25.0 ± 37.9
	Median [IQR]	23 [7–57]	8 [2-31]
	Range	1–348	1- 329
Individual specialist visits or requested diagnostic/laboratory tests	Mean ± SD	51.8 ± 76.1	27.2 ± 34.9
	Median [IQR]	25 [9–58.5]	15 [4-36]
	Range	1–779	1–359
Presence of previous hospitalizations and exemption codes, any time prior the ID			
Previous hospitalizations—N (%)		139 (26.1)	785 (14.9)
Prior assignment of exemption codes—N (%)		186 (34.9)	2,082 (39.6)

Abbreviations: ID: Index Date; SD: Standard Deviation; IQR: Interquartile Range.

Legend: *Chronic comorbidities (evaluated any time prior to ID); ^#^Up to 10 controls were matched for each acromegaly case by date of birth (± 2 years) and gender. For each matched control, the same ID of the corresponding matched case was assigned.

As for the univariable analysis, the potential predictors most strongly associated with acromegaly are shown in Supplementary Table 2.

**Figure 1:**
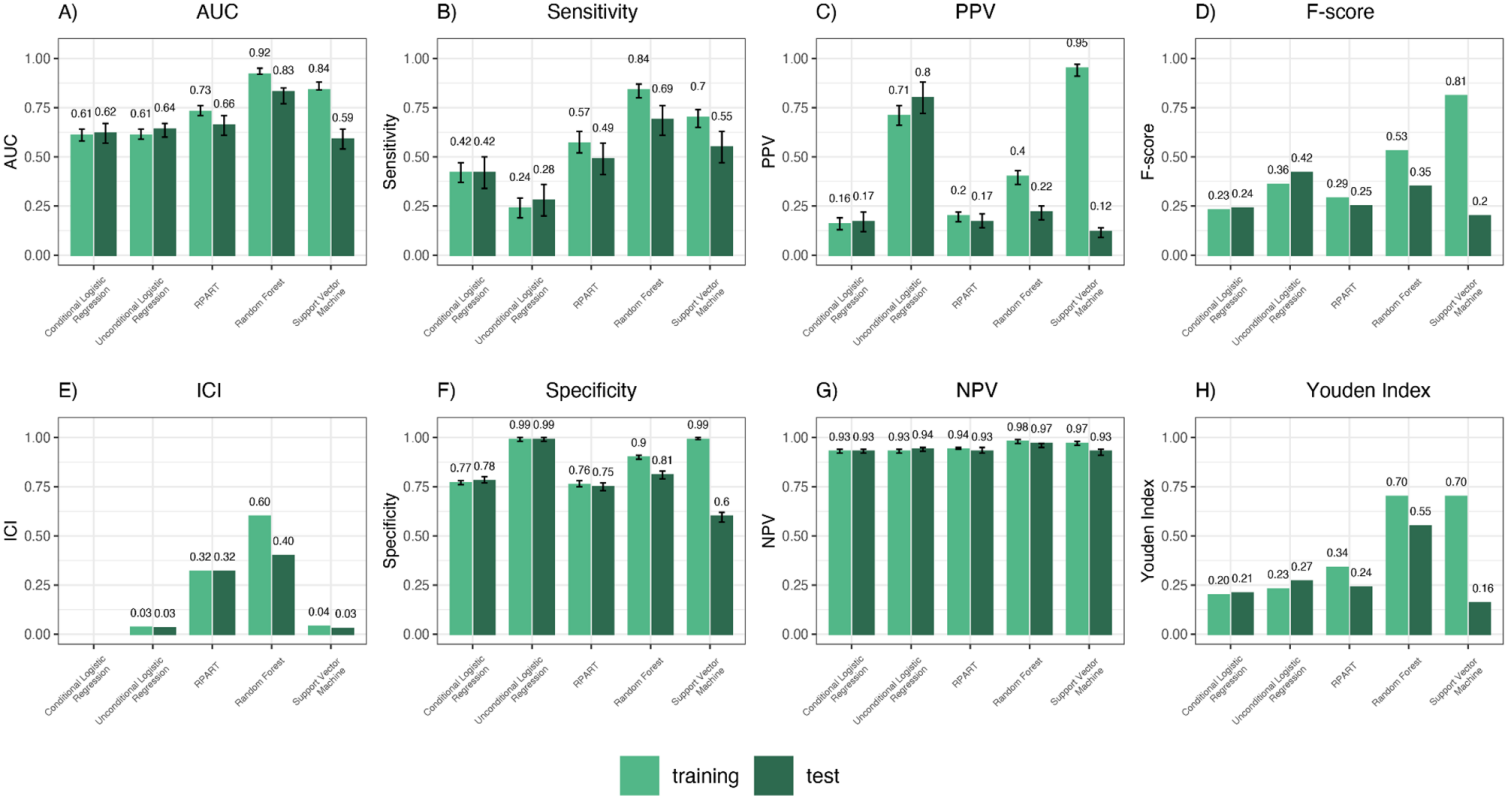
Performance of machine-learning algorithms for acromegaly diagnosis prediction, both in training and test sets, within 2 years prior to the index date. Abbreviations: AUC = area under the receiver operating characteristic curve; PPV = positive predictive value; ICI = integrated calibration index; NPV = negative predictive value; RPART = Recursive PArtitioning and Regression Tree; *Note:* Only the performances of the probabilistic version of predictive algorithms areshown. Sensitivity, Specificity, PPV, NPV, F-score and Youden Index were computed at the optimal threshold of predicted probabilities detected in the ROC curve space.

As for the machine-learning analysis, the training set included 373 cases and 3,676 controls, while the test set included 160 cases and 1,579 controls. Overall, the probabilistic RF achieved the highest discriminatory power in the test set, with an AUC of 0.83 (95% CI 0.79–0.87), followed by the RPART (AUC = 0.66, 95% CI 0.61–0.71), the unconditional logistic regression model (AUC = 0.64, 95% CI 0.60–0.67), the probabilistic SVM (AUC = 0.59, 95% CI 0.53–0.64) and the CLOGIT (AUC = 0.62, 95% CI 0.57–0.67). When subjects were classified according to the optimal threshold of their predicted probabilities in the test set, models’ sensitivity ranged from 28% for the unconditional logistic regression model to 69% for the RF, while the specificity ranged from 60% for the probabilistic SVM to 99% for the unconditional logistic regression model (Fig. 1). Furthermore, the probabilistic RF achieved the highest classification accuracy (Youden index = 0.35). The number of predictors selected by the algorithms was: 5 for the unconditional logistic regression model, 12 for the CLOGIT, 10 for the RPART, 38 for the probabilistic RF and 14 for the probabilistic SVM. Among the 38 predictors identified by the probabilistic RF model, which yielded the highest diagnostic accuracy, the most important 10 ones according to the relative variable importance (RVIMP) were: the presence of co-payment exemptions codes related to hypertensive disease [i.e., hypertension with organ damage (RVIMP: 100%) and hypertension without organ damage (RVIMP: 84.3%)], permanent disability (RVIMP: 83.0%), glaucoma (RVIMP: 64.5%), inflammatory bowel diseases [i.e., ulcerative colitis and Crohn disease (RVIMP: 61.4%)] and chronic hepatitis (RVIMP: 55.2%); the number of pharmacy claims related to immunosuppressants (RVIMP: 84.8%); the presence of diabetes (RVIMP: 60.1%) as comorbidity; the request for chest CT scan (RVIMP: 59.8%) and routine chest radiography (RVIMP: 58.3%). Algorithms resulted well calibrated (ICI values in test set ranged from 0.03 for unconditional logistic regression model and SVM to 0.40 for the RF).

The full list of predictors selected by each algorithm, along with the classification rule that can be used to predict the presence of acromegaly, is shown in Table 2.

**Table 2:** List of features (i.e., predictors) selected by each algorithm, along with the classification rule to predict the presence of acromegaly.

Cross-Validated multivariable conditional logistic regression with LASSO penalty	
Selected predictors	Formula
Number of pharmacy claims [II level ATC code] Diuretics [C03], Agents acting on the renin-angiotensin system [C09], Lipid-modifying agents [C10], Thyroid therapy [H03], Antineoplastic agents [L01], Immunosuppressants [L04], Psychoanaleptics [N06], Drugs for obstructive airway diseases [R03] Number of specialist visits or laboratory/diagnostic tests [code] General medical examination [89.7], Creatinine [90.16.3], Urea [90.44.1], Venous blood sampling [91.49.2]	Linear Predictor (LP) = 0.02965 × [C09] -0.01040 × [C10]—0.00362 × [C03] + 0.29712 × [L04] + 0.00432 × [N06] + 0.01216 × [R03] + 0.09161 × [89.7] + 0.03592 × [90.16.3] + 0.05474 × [90.44.1] + 0.05284 × [91.49.2] + 0.00538 × [H03] + 0.00390 × [L01] Classification rule: (1) for a new subject, collect all required variables and compute the formula. If there are no claims for a specific variable (or if this information is not available), the value of this variable must be set to 0; (2) if LP > 0.51 then the subject is classified as having the acromegaly disease; not otherwise (Sensitivity: 42%, Specificity: 78%)
Cross-Validated multivariable unconditional logistic regression with LASSO penalty	
Selected predictors	Formula
Number of pharmacy claims [II level ATC code] Immunosuppressants [L04] Number of specialist visits or laboratory/diagnostic tests [code] Corticotropin [90.15.2], Cortisol [90.15.3], Diagnoses [ICD-9 CM codes] Cardiomyopathy [425.xx], Benign neoplasm of pituitary gland and craniopharyngeal duct [227.3]	Estimated probability (P) = 1/{1 + exp-(-2.35080 + 0.77800 × [425.xx] + 0.02573 × [227.3] + 0.16050 × [L04] + 0.64099 × [90.15.2] + 0.55470 × [90.15.3])} Classification rule: (1) for a new subject, collect all required variables and compute the formula. If there are no claims for a specific variable (or if this information is not available), the value of this variable must be set to 0 (2) if *P* > 0.09 then the subject is classified as having the acromegaly disease; not otherwise (Sensitivity: 28%, Specificity: 99%)
Case-weighted (IPW) Recursive PArtitioning and Regression Tree (RPART)	
Selected predictors	Classification Tree
Number of pharmacy claims [II level ATC code] Immunosuppressants [L04], Antibacterials for systemic use [J01], Agents acting on the renin-angiotensin system [C09], Antiinflammatory and antirheumatic products [M01] Diagnoses [ICD-9 CM codes] Diabetes [250.xx] Number of specialist visits or laboratory/diagnostic tests [code] Free thyroxine [90.42.3], Chest CT scan [87.41.1], Electrocardiogram [89.52], Total cholesterol [90.14.3], Cortisol [90.15.3]	
Case-weighted (IPW) Probabilistic Random Forest	
Selected predictors	Relative variable importance (%)^§^
Number of pharmacy claims [II level ATC code] Immunosuppressants [L04], Thyroid therapy [H03], Immunostimulants [L03], Endocrine therapy [L02], Digestives, including enzymes [A09], Bile and liver therapy [A05], Antihemorrhagics [B02], Diuretics [C03], Antigout preparations [M04] Diagnoses [ICD-9 CM codes] Diabetes [250.xx], Cardiomyopathy [425.xx], Other chronic non-alcoholic liver disease [571.8], Calculus of gallbladder without mention of cholecystitis [574.20] Co-payment exemptions [code] Hypertensive heart disease with organ damage [031] Hypertensive heart disease without organ damage [0A31], Co-payment exemption for disability [C03], Glaucoma [019], Ulcerative colitis and Crohn disease [009], Chronic hepatitis [016], Familiar hypercholesterolemia [025], Work disability [L02], Psoriasis [045], Malignant neoplasms [048] Systemic lupus erythematosus [028], Celiac disease [I0060] Number of specialist visits or laboratory/diagnostic tests [code] Routine chest radiography [87.44.1], Chest CT scan [87.41.1], Abdominal ultrasonography [88.76.1], Thyroid and parathyroid ultrasound [88.71.4], CT of abdomen with or without contrast agents [88.01.6], Free thyroxine [90.42.3], Free triiodothyronine [90.43.3], Disabilities secondary to degenerative osteomioarticular diseases [93.60.01], Thoraco-dorsal spine X-ray [87.23], Lumbosacral x-ray [87.24.1], Thyrotropin [90.42.1], Pace-maker control visit [89.48.1], Visual field examination [95.05]	^§^Only the first 15 predictors are shown Legend: * Co-payment exemptions ^ Specialist examinations or laboratory tests # Diagnoses ° Pharmacy claims
Case-weighted (IPW) Probabilistic Support Vector Machine	
Selected predictors	Relative variable importance (%)
Number of pharmacy claims [II level ATC code] Antibacterials for systemic use [J01], Drugs for acid-related disorders [A02], Immunosuppressants [L04] Number of specialist visits or laboratory/diagnostic tests [code] Faecal occult blood [90.21.4], X-ray of bones and joints [87.29], Free thyroxine [90.42.3], Alkaline phosphatase [90.23.5], Esophagogastroduodenoscopy [45.13], Electrocardiogram [89.52], Colonoscopy [45.23], Total calcium [90.11.4], Urate [90.43.5] Co-payment exemptions [code] Co-payment exemption for disability [C03], Hypertensive heart disease without organ damage [0A31]	Legend: * Co-payment exemptions ^ Specialist examinations or laboratory tests # Diagnoses ° Pharmacy claims

Abbreviations: RA = renin-angiotensin; CT = computed tomography;

^§^Only the first 15 predictors are shown.

The optimal values set for tuning parameters and thresholds for each predictive model and algorithm are shown in Supplementary Table 3.The structure of the R code used to perform machine learning algorithms is shown in Supplementary Document 3.

**Figure 2:**
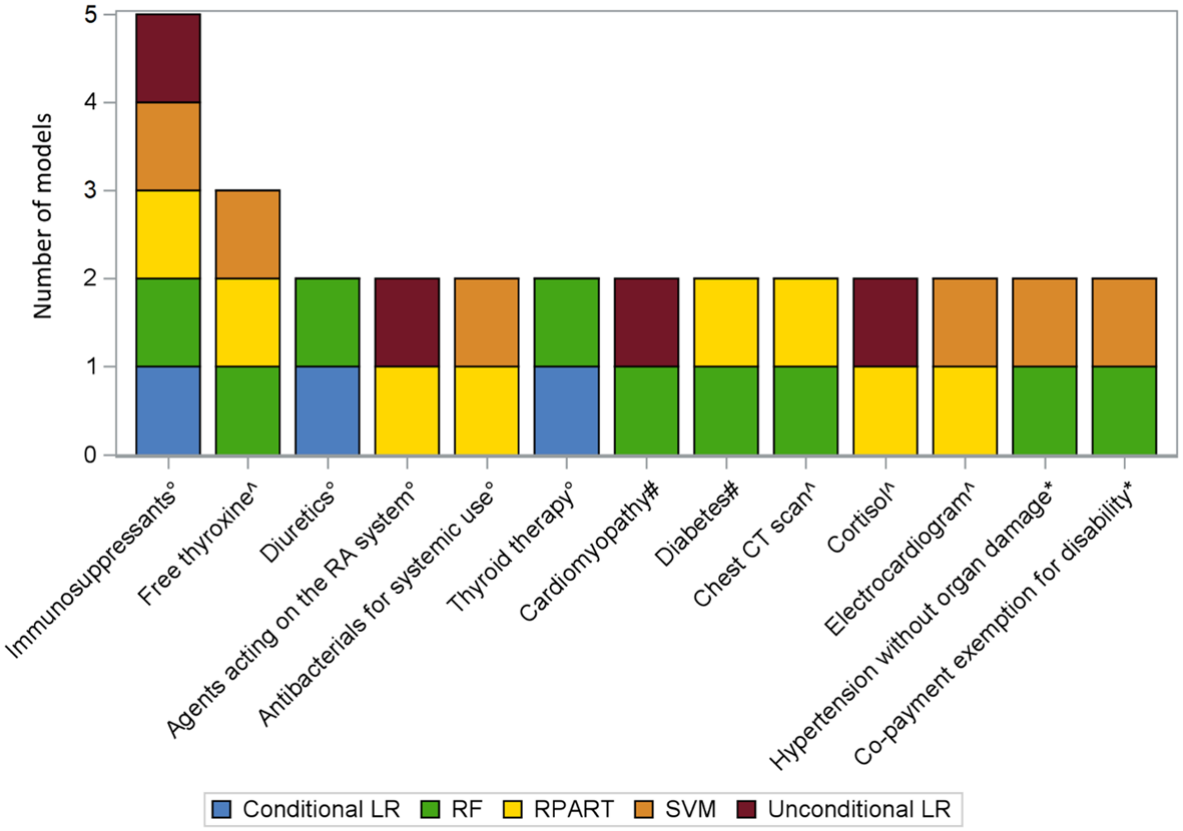
Stacked bar chart showing the frequency distribution of the acromegaly predictors identified by more than one predictive algorithm. Abbreviations: CT = computed tomography; LR = logistic regression; RA = renin-angiotensin; RF = probabilistic random forest; RPART = recursive partitioning and regression tree; SVM = probabilistic support vector machine. Legend: * Co-payment exemptions, ^ Specialist examinations or lab tests, # Diagnoses, ° Pharmacy claims.

Overall, the only diagnosis predictor selected by all five algorithms was the number of immunosuppressants-related pharmacy claims (II level ATC: L04). The other diagnosis predictors selected by at least two models were: the number of pharmacy claims related to agents acting on the renin-angiotensin system (II level ATC: C09), diuretics (II level ATC: C03), antibacterials for systemic use (II level ATC: J01) and thyroid therapy (II level ATC: H03); the presence of cardiomyopathy and diabetes as comorbidities; the presence of co-payment exemption codes related to permanent disability and hypertensive disease without organ damage; the request for chest CT scan, electrocardiogram, cortisol level dosing, and free thyroxine level dosing (Fig. 2).

The total frequency, number of claims and the mean number of claims per subject of each predictor identified by more than one predictive algorithm are shown in Table 3.

**Table 3:** Total number of subjects (frequency) and claims of each predictor identified by more than one predictive algorithm, among cases and matched controls.

Predictors selected by at least 2 algorithms	Cases (N = 533)			Controls (N = 5,255)			Mean ratio° (95% CI)	Odds ratio^#^ (95% CI)
	N. patients (%)	N claims	Mean number of claims per subject*	N. patients (%)	N claims	Mean number of claims per subject*		
Number of pharmacy claims related to immunosuppressant drugs (II level ATC: L04)	75 (14.1)	588	7.84	24 (0.5)	135	5.63	1.39 (0.92–2.12)	–
Free thyroxine level measurement	132 (24.8)	238	1.80	507 (9.6)	948	1.87	0.96 (0.82–1.13)	–
Number of pharmacy claims related to diuretics (II level ATC: C03)	80 (15.0)	318	3.98	388 (7.4)	2,241	5.78	0.69 (0.49–0.98)	–
Number of pharmacy claims related to agents acting on the renin-angiotensin system (II level ATC: C09)	175 (32.8)	1,678	9.59	1,338 (25.5)	11,862	8.87	1.08 (0.95–1.23)	–
Number of pharmacy claims related to antibacterials for systemic use (II level ATC: J01)	252 (47.3)	972	3.86	2,370 (45.1)	7,175	3.03	1.27 (1.12–1.45)	–
Number of pharmacy claims related to thyroid therapy (II level ATC: H03)	71 (13.3)	387	5.45	255 (4.9)	1,464	5.74	0.95 (0.75–1.20)	–
Diagnosis of cardiomyopathy	17 (3.2)	17	1.00	8 (0.2)	8	–	–	23.52 (9.74–56.82)
Diagnosis of diabetes	118 (22.1)	118	1.00	817 (15.5)	817	–	–	1.60 (1.28–2.02)
Chest CT scan	34 (6.4)	56	1.65	50 (1.0)	83	1.66	0.99 (0.70–1.41)	–
Cortisol level measurement	36 (6.8)	67	1.86	15 (0.3)	18	1.20	1.55 (0.50–4.82)	–
Electrocardiogram	93 (17.4)	126	1.35	547 (10.4)	801	1.46	0.93 (0.81–1.06)	–
Co-payment exemption for hypertension without organ damage	55 (10.3)	55	1.00	638 (12.1)	638	–	–	0.80 (0.60–1.10)
Co-payment exemption for disability	14 (2.6)	14	1.00	127 (2.4)	127	–	–	0.98 (0.56–1.74)

Abbreviations: ATC = anatomical therapeutic chemical classification; CT = computed tomography; 95% CI = 95% confidence interval.

*This measure is calculated as the ratio of the total number of (code) claims to the number of subjects with at least one claim; °This measure is calculated as the ratio of the mean number of claims per subject between cases and controls and indicates how many times the mean number of claims per subject in cases is higher than in matched controls and was estimated by over dispersed Poisson model; ^#^Odds ratio from conditional logistic regression models.

**Figure 3:**
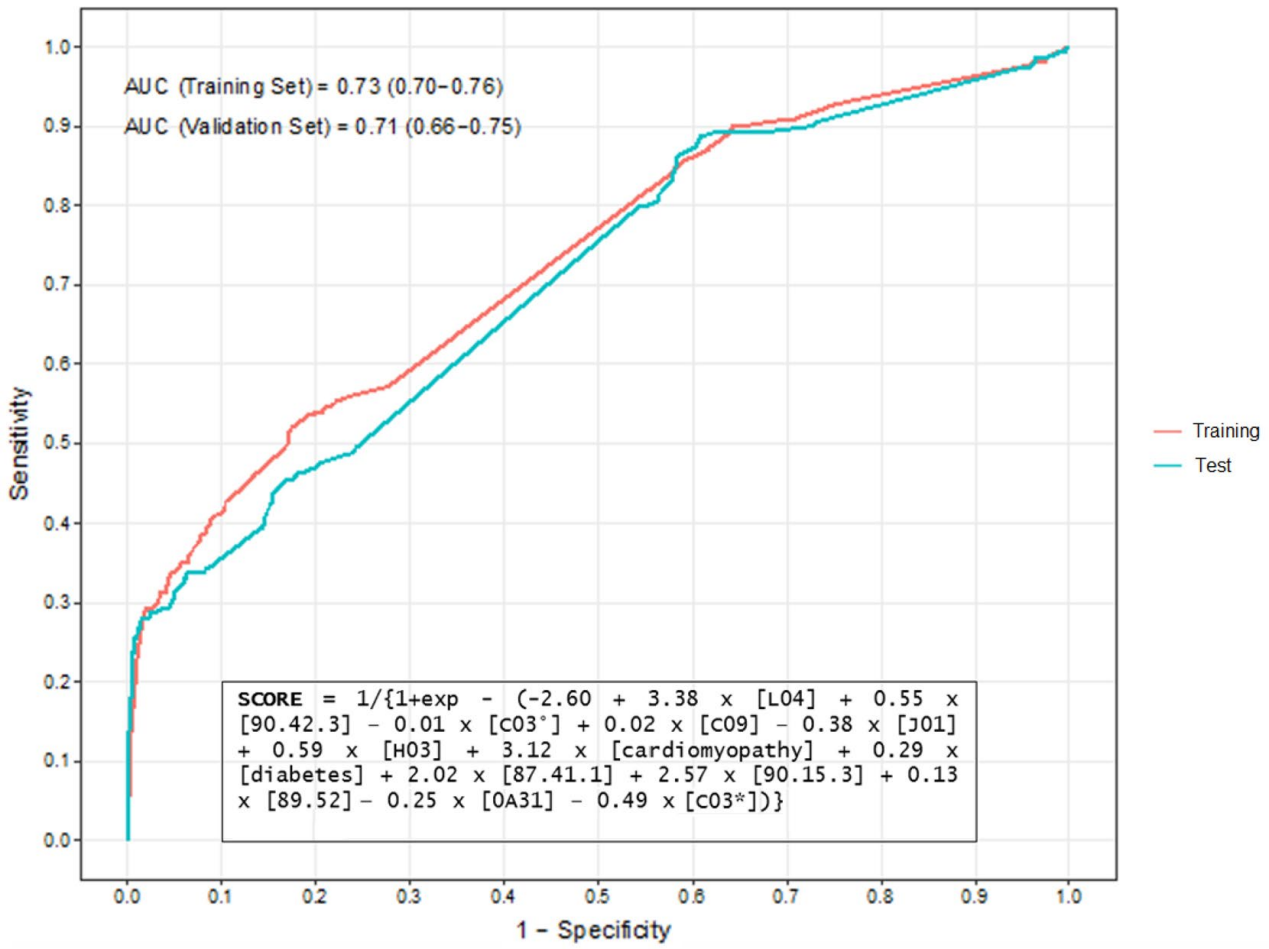
Receiver Operator Characteristic curve of the predicted individual probabilities computed by the multivariable logistic regression model used to develop the meta-score for the prediction of the diagnosis of acromegaly. Note: all predictors included in this formula were dichotomised (i.e., presence/absence of the specific condition). Legend: L04 = number of pharmacy claims related to immunosuppressants; 90.42.3 = request for free thyroxine level measurement; C03° = number of pharmacy claims related to diuretics; C09 = number of pharmacy claims related to agents acting on the renin-angiotensin system; J01 = number of pharmacy claims related to antibacterials for systemic use; H03 = number of pharmacy claims related to thyroid therapy; 87.41.1 = request for computed tomography of chest; 90.15.3 = request for cortisol level measurement; 89.52 = request for electrocardiogram; 0A31 = co-payment exemption code related to hypertensive disease without organ damage; C03* = co-payment exemption codes related to permanent disability.

The predictors selected by ≥ 2 algorithms (13 features) were used to develop the meta-score, which yielded an AUC equal to 0.71 (95% CI 0.66–0.75) in the test set (Fig. 3).

The continuous predictors included in the meta-score were dichotomized because it was found that the model achieved a higher AUC than the one that included the original predictors. The optimal threshold value of this score, above which physicians should consider performing further investigations to assess the presence of acromegaly, was found to be equal to 0.08, achieving low sensitivity (40%) but high specificity (80%). In particular, the variables mostly associated with the diagnosis of acromegaly according to the meta-score were the number of immunosuppressants-related pharmacy claims, the presence of cardiomyopathy as comorbidity and the requests for chest CT scan and cortisol level measurement.

The performance of any machine-learning algorithm at different sensibility and specificity thresholds was reassessed after correction for “misclassification” and results were virtually consistent with the original ones (see Supplementary Document 2).

**Figure 4:**
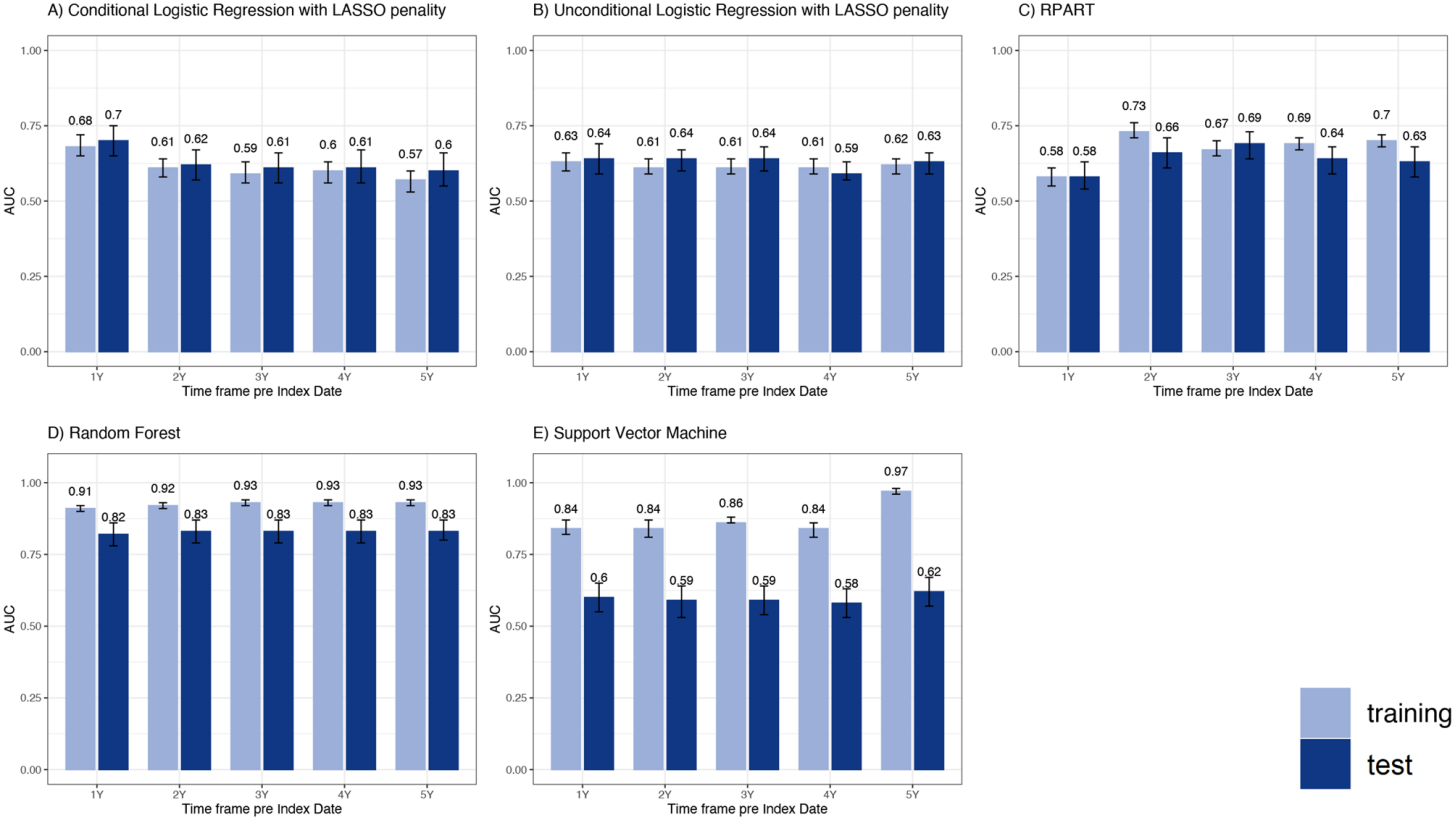
Performance of machine-learning algorithms for acromegaly diagnosis prediction, both in training and in test sets, within 1 to 5 years prior to the index date. Abbreviations: AUC** = **area under the receiver operating characteristic curve; LASSO = Least Absolute Shrinkage and Selection Operator; RPART = Recursive Partitioning and Regression Tree. Note: Only the performances of the probabilistic version of predictivealgorithms are shown. Sensitivity, Specificity, PPV, NPV, F-score and Youden Index were computed at the optimal threshold of predicted probabilities detected in the ROC curve space.

Concerning the sensitivity analysis, the algorithm yielding the highest AUC values in the test set for each timeframe was the probabilistic RF, that were almost the same for each considered timeframe (from 0.82 within the 1 year prior to ID timeframe to 0.83 in the all the other timeframes) (Fig. 4).

## Discussion

To our knowledge, this is the first population-based study that applied traditional statistical models and machine learning algorithms to identify a combination of predictive variables for the early diagnosis of acromegaly using administrative claims databases.

All the five predictive algorithms achieved poor diagnostic accuracy, except for RF that yielded an excellent discriminatory power, as shown by the AUC values in the test set. Nevertheless, the meta-score developed by using an unconditional multivariable logistic regression model including the predictors selected by at least two algorithms achieved acceptable discriminatory power. In general, the proposed algorithms achieved consistent results in both training and test sets, except for the probabilistic SVM, for which considerable discrepancies were observed, mainly concerning PPV, F-score, Youden index, AUC, and specificity. To explain such performance discrepancies, it is important to mention that the large number of variables included may lead to an SVM classifier’s overfitting (i.e., the phenomenon by which a learning machine loses its learning generalization capability in classification), providing deceptive diagnostic results^25^. Therefore, although the probabilistic SVM yielded good diagnostic performances in training data, it was not able to generalize the diagnostic ability in the test set. In contrast, the unconditional logistic regression model performed slightly better in the test set than in the training set, but this difference was not statistically significant. This can be explained by the ability of regression models to predict the response to an input that lies outside the range of values of the predictor variable used to fit the model (i.e., extrapolation)^26,27^.

Overall, except for probabilistic SVM, machine learning algorithms yielded better performances in terms of AUC and sensitivity as compared to logistic regression models, while the latter performed better in terms of specificity and PPV.

In the field of rare diseases, two studies developed and tested different claims-based machine learning algorithms for the early diagnosis of pulmonary hypertension. Confirming our findings, both studies showed that, as compared to other machine learning algorithms, the RF yielded the best diagnostic performances.

Machine learning methods encompass a wide range of different algorithms that can either (i) model nonlinear relationships, resulting in complex “black box” that are hard to understand because it is very difficult to explore how variables are combined to make predictions, or (ii) simultaneously perform variable selection and produce clinically interpretable solutions (e.g., logistic regression models with LASSO penalty), which return a classification rule based on the individual linear weighted combination of included predictors.

Overall, most of the predictors identified by each algorithm for the early diagnosis of acromegaly were selected from the diagnostic tests and specialist’s visits database (N = 33) and pharmacy claims database (N = 25). The only predictor selected by all five algorithms was the number of pharmacy claims for immunosuppressants, thus potentially suggesting that the presence of systemic inflammation may be one of the key predictors for the early diagnosis of acromegaly^28^. Indeed, several case reports published in the literature describe patients concomitantly affected by acromegaly and immune-mediated diseases, including rheumatoid arthritis^29–32^, ulcerative colitis^33,34^, psoriasis^35–39^, myasthenia gravis^40–42^, as well as anti-neutrophil cytoplasmic antibodies (ANCA)-associated vasculitis and Sjögren’s Syndrome^43^.

One of the main strengths of this study is the large sample size, with a total of more than 5,000,000 patients, which is particularly important for research in the field of rare diseases, where the number of affected patients is very small. Furthermore, the use of a validated coding algorithm for the identification of acromegalic patients in claims databases, yielding high diagnostic performances, minimized the risk of misclassification.

However, some limitations are worth mentioning. First, the predictor-diagnosis relationships discovered from data driven approaches, such as machine learning algorithms, do not always imply a causal relationship; however, the development of a meta-score allowed us to obtain a clinically interpretable classification rule which could be helpful for the early diagnosis of acromegaly, although it has a lower diagnostic accuracy as compared to the RF. Second, since claims databases do not allow tracking health services purchased privately by citizens and socio-health activities (e.g., admissions to residences) and the absence of this information may have prevented the identification of some potential predictors of acromegaly diagnosis. However, considering that acromegaly is mainly managed in the specialist setting, this has unlikely affected the findings of this study. Third, considering that the diagnostic predictive algorithms have been applied on claims databases, this study presents some limitations related to this type of data sources, such as the presence of missing values and the potentially inaccurate coding practice. As a result, the ID used to define the timeframes for the diagnostic prediction may have been misclassified and, as such, it could not exactly coincide with the actual date of the first diagnosis of acromegaly. Consequently, it is possible that some of the potential predictors identified by the different algorithms should be considered as treatment-related variables rather than predictors of the early diagnosis of acromegaly. As an example, features selected by the RF include the number of pharmacy claims related to bile and liver therapy and the diagnosis of calculus of gallbladder, which are likely due to somatostatin analogues therapy^44,45^. Nevertheless, it should be noted that these features were selected only by one of the five proposed algorithms and that they were not among the first 15 selected features in terms of RVIMP.

## Conclusions

In this study we developed and internally validated machine-learning algorithms for the early diagnosis of acromegaly using administrative claims databases. Findings showed that data-driven machine learning algorithms can play a role in predicting the diagnosis of rare diseases such as acromegaly. Of the five predictive algorithms developed, only the RF yielded an excellent discriminatory power, while the others achieved poor diagnostic accuracy and the meta-score developed on the predictors selected by at least two algorithms achieved an acceptable accuracy. The predictor mostly associated with the presence of acromegaly was the number of pharmacy claims related to immunosuppressants, potentially suggesting that systemic inflammation and/or autoimmune diseases may be key predictors of acromegaly diagnosis.

### Supplementary Information

Supplementary Information.

## Data Availability

The data that support the findings of this study are available from Sicily Region, but restrictions apply to the availability of these data, which were used under license for the current study, and so are not publicly available. Data are however available from the authors upon reasonable request and with permission of Sicily Region.
