# Supplementary material for: Machine learning-based algorithms applied to drug prescriptions and other healthcare services in the Sicilian claims database to identify acromegaly as a model for the earlier diagnosis of rare diseases
Source: Sci Rep. 2024 Mar 14;14:6186. doi: 10.1038/s41598-024-56240-w (PMC10940660; doi:10.1038/s41598-024-56240-w)
Supplement: Supplementary file 1 — Supplementary Information. [file 41598_2024_56240_MOESM1_ESM.docx]

Supplementary Material

**Supplementary Table 1.** List of ICD-9-CM, exemption codes and ATC codes used to identify comorbidities.

| **Comorbidity** | **ICD9-CM Codes** | **Co-payment exemption codes** | **ATC codes** |
| --- | --- | --- | --- |
| Inflammatory bowel diseases | 555.x, 556.x | 009.555, 555, 009.556, 556 | L04AA33 |
| Colon polyp | 211.3 | - | - |
| Colon cancer | 153.x, 154.0, 197.5 | - | - |
| Rheumatoid arthritis | 714.x | 006.714, 714 | - |
| Osteoarthritis | 715.xx | - | - |
| Osteoporosis | 733.00, 733.02, 733.03 | - | M05B* |
| Arthropathy/arthralgia/synovitis | 713.x, 716.4x-716.5x, 716.8x-716.9x, 719.4x, 727.0x | - | - |
| Carpal tunnel syndrome | 354.0 | - | - |
| Multiple sclerosis | 340 | 046 | L04AA23, L04AA27, L04AA31 |
| Prophylaxis of organ rejection | V42.x, 996.8 | - | L04AD02, L04AA06 |
| Cardiomyopathy | 425.x | - | - |
| Cardiac hypertrophy | 429.3 | - | - |
| Heart failure | 402.01, 402.11, 402.91, 404.01. 404.03, 404.11, 404.13, 404.91, 404.93, 428.xx | 021.428, 021, 428 | - |
| Cardiac dysrhythmia/arrhythmia | 427.xx | 0A02.427 | C01B* |
| Cerebrovascular diseases | 430.xx -438.xx | - | - |
| Diabetes mellitus | 250.xx | 013 | A10* |
| Sleep Apnea | 327.20, 327.21, 327.23, 327.26-327.29, 780.51, 780.53, 780.57 | - | - |
| Chronic kidney disease | 585.xx | 023 |  |
| Galactorrhea | 611.6, 676.6x | - | - |
| Menstrual abnormality | 626.x, 627.0-627.1 | - | - |
| Impaired libido/impotence | 302.72, 607.84, 799.81 | - | - |
| Hypopituitarism | 253.2, 253.7 | - | - |

**Abbreviations:** ICD-9-CM: International Classification of Diseases - 9th revision - Clinical Modification; ATC: Anatomical Therapeutic Chemical classification system

**Supplementary Figure 1.** Overall distribution of the time elapsed between the first record’s registration at each data source and the index date in all acromegaly subjects (cases) and matched controls.

**
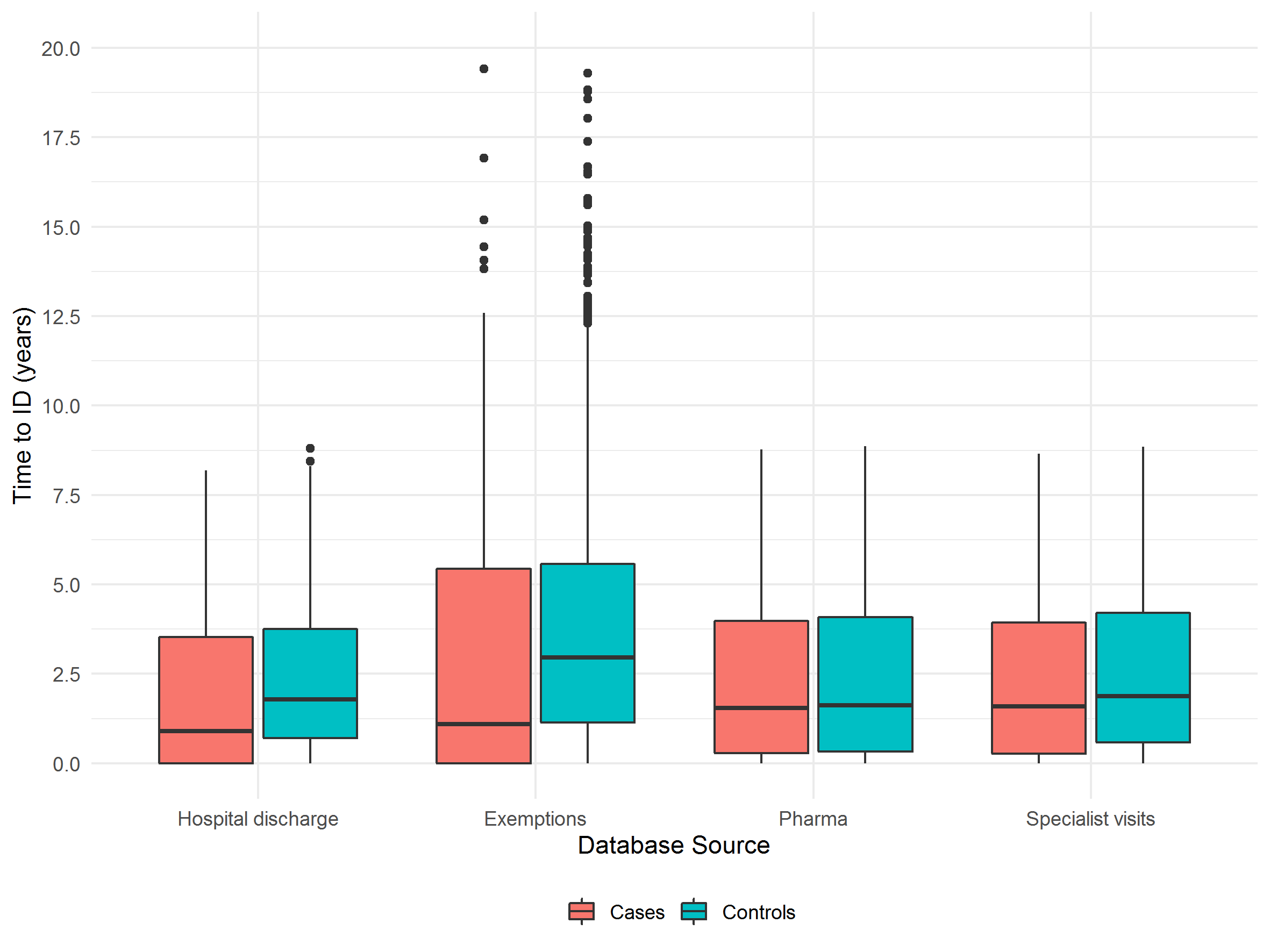
**

**Supplementary Document 1.** Details and peculiarities of the models and machine learning algorithms used for the early diagnosis of acromegaly.

**Cross-validated multivariable logistic regressions with LASSO penalty**

In any penalized multivariable model, the application of a “penalty” is intended to shrink the values of the regression coefficient of the less contributive variables towards zero. This results in a natural selection of predictors that produce an optimal weighted linear combination with respect to a given penalty parameter. In our case, the strength of using a penalized multivariable logistic regression model is its interpretive simplicity and the possibility of expressing the relationship between predictors and disease through a simple mathematical formula. Features were automatically selected according to a sort of backward selection procedure, with the aim of minimizing the sum of the negative binomial log-likelihood (i.e. likelihood of the data, also known as “binomial deviance”) and the L1 penalty function (also known as “LASSO” penalty), which is defined as the linear combination of the absolute values of the regression coefficients (i.e. betas), weighted for the given penalty parameter (lambda). The optimal value of the lambda parameter was identified in a grid search as the one that minimizes the mean of the previous objective function (i.e. binomial deviance + LASSO penalty) achieved after a 10-fold Cross Validation (CV) procedure. The reason why the unconditional logistic regression was performed in addition to the conditional LASSO model (CLOGIT) is that, although the CLOGIT is the most appropriate in this context (due to matching), the unconditional model allows the computation of individual predicted probabilities, whereas the CLOGIT only allows the computation of the linear predictor (LP) (i.e., the linear combination of selected predictors). Thus, the AUC achieved from CLOGIT can only be calculated on the LP scores, as it was not possible to convert these into predicted probabilities. Therefore, in CLOGIT it is possible to assign a binary classification of the subject with respect to the optimal LP threshold value identified by the ROC curve.

**Tree-based algorithms (RPART and RF)**

RPART is a decision tree that recursively splits the data in subgroups, based on the values of the input variables (all the features), with the goal of maximizing the homogeneity of the target variable within each subgroup. Features were automatically selected hierarchically (thus defining the tree nodes), having set a priori that at least 50 observations are required at each node of the tree to split it further, resulting in a large unpruned tree (i.e. the stopping rule). To reduce the number of selected features (i.e. tree pruning step), a “complexity” parameter (cp) that penalizes the tree as the number of splits increases was imposed. The cp is defined as the minimum improvement in misclassification error needed at each node. The higher the cp, the smaller the tree. The optimal value of this cp was determined in a grid search as that which the 10-fold CV classification error was minimized.

RF is a popular machine learning algorithm that consists in an ensemble of decision trees, where each tree is built on a random set of candidate variables. In the probabilistic version of the RF, each decision tree estimates the probability of having the disease and all of these estimates are finally averaged to get an estimate of the “probability of the forest”. To achieve the highest stability of individual probability prediction, it was set *a priori* that the forest includes a very large number of trees (100,000 trees). The node size of the final leaves of each tree was identified as the one that produced the highest AUC in the test set (choosing among 50, 150, 300 and 500 inverse probability weighted observations per node). In both cases, the OOB data are also used to obtain a variable importance (VIMP) estimate: this is defined as the average over all trees of the difference in prediction error before and after the permutation of the values of each variable in the OOB data within each tree. The relative VIMP (RVIMP) standardizes the importance values for ease of interpretation and is defined as the percent improvement with respect to the most important predictor. For sake of simplicity, only those features that reached at least 5% of the RVIMP were shown. RF do not perform a proper “feature selection” (as all possible covariates are always exploited within these algorithms) but merely rank them from most to least important. As the number of cases was extremely lower than the number of controls, to account for these imbalances and increase the accuracy in correctly predicting the probability of detecting cases, different weights were allocated to cases and controls, only in the training set, when running these two tree-based algorithms, following the inverse probability weight (IPW) method.

**Support Vector Machine**

SVM is a supervised machine learning algorithm allowing to solve binary classification issues, classifying both linear and non-linear data. It uses kernel functions to identify elements in a space without using coordinates but calculating the inner product between the images of all data pairs in the function space. This operation, called kernel trick, is often computationally cheaper than the coordinate calculation. The most used kernel functions are the linear, radial and the polynomial ones. SVM aims at generating a hyperplane with the main purpose to optimize the distance between the nearest points belonging to two different classes, known as support vectors. In our study, the value of the cost (C) and gamma parameters was chosen following a 10-fold cross validation of the training set. These values were determined as the ones that maximize the accuracy. Also SVM do not perform a proper “feature selection” (as all possible covariates are always exploited within these algorithms) but merely rank them from most to least important. For sake of simplicity, only those features that reached at least 5% of the RVIMP were shown.

**Supplementary Table 2.** Counts of pharmacy claims and specialist visits or laboratory/diagnostic tests, evaluated within two years prior the index date, between patients affected by acromegaly (cases) and matched controls.

|  | **Code** | **Description** | **N_subj_ - N(%)** | | **N_reg_/N_subj_** | | **Mean ratio among subjects with at least one registration^#^** | | **N_reg_/all subjects** | | **Mean ratio among all subjects^#^** | |
| --- | --- | --- | --- | --- | --- | --- | --- | --- | --- | --- | --- | --- |
|  |  |  |  |  | **(mean number per subject)*** | |  |  | **(mean number per subject)^°^** | |  |  |
|  |  |  | **Cases (N=533)** | **Controls (N=5,255)** | **Cases** | **Controls** | **Mean ratio (95%CI)** | **p-value** | **Cases** | **Controls** | **Mean ratio (95%CI)** | **p-value** |
| **Pharmacy claims (2^nd^ level ATC)** | L04 | Immunosuppressants | 75 (14.1) | 24 (0.5) | 588/75 (7.8) | 135/24 (5.6) | 1.39  (0.92-2.12) | 1.2x10^-1^ | 588/533 (1.1) | 135/5,255  (0.0) | 42.94  (22.73-81.13) | 6.8x10^-29^ |
|  | H01 | Pituitary and hypothalamic hormones and analogues | 3 (0.6) | 3 (0.1) | 49/3 (16.3) | 18/3 (6.0) | 2.72  (0.61-12.11) | 2.62x10^-1^ | 49/533 (0.1) | 18/5,255  (0.0) | 26.84  (4.95-145.42) | 8.5x10^-03^ |
|  | L03 | Immunostimulants | 19 (3.6) | 9 (0.2) | 85/19 (4.5) | 47/9 (5.2) | 0.86  (0.46-1.61) | 6.4x10^-1^ | 85/533  (0.2) | 47/5,255  (0.0) | 17.83  (6.08-52.31) | 1.0x10^-05^ |
|  | A04 | Antiemetics and antinauseants | 11 (2.1) | 8 (0.2) | 34/11 (3.1) | 27/8 (3.4) | 0.92  (0.30-2.80) | 8.8x10^-1^ | 34/533 (0.1) | 27/5,255  (0.0) | 12.42  (3.52-43.74) | 5.5x10^-03^ |
|  | L01 | Antineoplastic agents | 24 (4.5) | 51 (1.0) | 318/24  (13.2) | 462/51 (9.1) | 1.46  (0.95-2.26) | 9.1x10^-2^ | 318/533  (0.6) | 462/5,255  (0.01) | 6.79  (3.65-12.60) | 8.8x10^-08^ |
|  | L02 | Endocrine therapy | 12 (2.3) | 25 (0.5) | 114/12  (9.5) | 205/25 (8.2) | 1.16  (0.61-2.20) | 6.6x10^-1^ | 114/533 (0.2) | 205/5,255  (0.0) | 5.48  (2.31-13.04) | 7.4x10^-03^ |
|  | J06 | Immune sera and immunoglobulins | 11 (2.1) | 42 (0.8) | 17/11 (1.5) | 46/42 (1.1) | 1.41  (0.96-2.07) | 8.6x10^-2^ | 17/533 (0.0) | 46/5,255  (0.0) | 3.64  (1.90-7.00) | 6.4x10^-03^ |
|  | H03 | Thyroid therapy | 71 (13.3) | 255 (4.9) | 387/71 (5.6) | 1,464/255 (5.7) | 0.95  (0.75-1.20) | 6.7x10^-1^ | 387/533 (0.7) | 1,464/5,255  (0.3) | 2.61  (1.83-3.70) | 5.9x10^-06^ |
|  | H02 | Systemic corticosteroids | 116 (21.8) | 703 (13.4) | 418/116  (3.6) | 1,630/703  (2.3) | 1.55  (1.24-1.94) | 1.1x10^-4^ | 418/533  (0.8) | 1,630/5,255  (0.3) | 2.53  (1.93-3.31) | 1.1x10^-09^ |
|  | A11 | Vitamins | 58 (10.9) | 296 (5.6) | 312/58  (5.4) | 1,609/296  (5.4) | 0.99  (0.76-1.29) | 9.4x10^-1^ | 312/533  (0.6) | 1,609/5,255  (0.3) | 1.91  (1.31-2.79) | 4.8x10^-02^ |
|  | C09 | Agents acting on the renin-angiotensin system | 175 (32.8) | 1,338 (25.5) | 1678/175  (9.6) | 11,862/1,338  (8.9) | 1.08  (0.95-1.23) | 2.4x10^-1^ | 1,678/533  (3.2) | 11,862/5,255  (2.3) | 1.39  (1.16-1.68) | 2.7x10^-02^ |
|  | J01 | Antibacterials for systemic use | 252 (47.3) | 2,370 (45.1) | 972/252  (3.9) | 7175/2,370  (3.0) | 1.27  (1.12-1.45) | 2.0x10^-4^ | 972/533  (1.8) | 7,175/5,255  (1.4) | 1.34  (1.14-1.56) | 1.5x10^-02^ |
| **Pharmacy claims (5^th^ level ATC)** | H02AB10 | Cortisone | 18 (3.4) | 2 (0.0) | 109/18 (6.1) | 2/2 (1.0) | 6.06  (0.13-293.25) | 3.7x10^-1^ | 109/533 (0.2) | 2/5,255  (0.0) | 537.33  (68.96-4186.73) | 1.2x10^-06^ |
|  | L04AB01 | Etanercept | 11 (2.1) | 1 (0.0) | 54/11 (4.9) | 1/1 (1.0) | 4.91  (0.15-161.11) | 3.9x10^-1^ | 54/533 (0.1) | 1/5,255  (0.0) | 532.40  (43.22-6558.84) | 5.9x10^-04^ |
|  | L04AA06 | Mycophenolic acid | 11 (2.1) | 1 (0.0) | 35/11 (3.2) | 4/1 (4.0) | 0.80  (0.14-4.52) | 8.0x10^-1^ | 35/533 (0.1) | 4/5,255  (0.0) | 86.27  (10.47-710.63) | 2.1x10^-02^ |
|  | C09BB07 | Ramipril and amlodipine | 2 (0.4) | 3 (0.1) | 24/2 (12.0) | 6/3 (2.0) | 6.00  (3.08-11.69) | 1.3x10^-2^ | 24/533 (0.1) | 6/5,255  (0.0) | 39.44  (7.86-197.84) | 4.9x10^-03^ |
|  | A04AA01 | Ondansetron | 9 (1.7) | 6 (0.1) | 30/9 (3.3) | 9/6 (1.5) | 2.22  (0.46-10.82) | 3.4x10^-1^ | 30/533 (0.1) | 9/5,255  (0.0) | 32.86  (9.90-109.06) | 7.3x10^-06^ |
|  | L01XA02 | Carboplatin | 7 (1.3) | 4 (0.1) | 32/7 (4.6) | 10/4 (2.5) | 1.83  (1.19-2.80) | 2.2x10^-2^ | 32/533 (0.1) | 10/5,255  (0.0) | 31.55  (8.94-111.36) | 5.1x10^-05^ |
|  | B03AA07 | Ferrous sulfate | 15 (2.8) | 54 (1.0) | 43/15 (2.9) | 89/54 (1.7) | 1.74  (1.10-2.75) | 2.1x10^-2^ | 43/533 (0.1) | 89/5,255  (0.0) | 4.76  (2.53-8.96) | 8.0x10^-04^ |
|  | B01AB05 | Enoxaparin | 29 (5.4) | 124 (2.4) | 84/29  (2.9) | 251/124 (2.0) | 1.43  (0.94-2.18) | 9.7x10^-2^ | 84/533 (0.2) | 251/5,255  (0.1) | 3.30  (1.96-5.55) | 4.3x10^-03^ |
|  | H02AB07 | Prednisone | 51 (9.6) | 246 (4.7) | 154/51  (3.0) | 488/246 (2.0) | 1.52  (1.09-2.13) | 1.4x10^-2^ | 154/533  (0.3) | 488/5,255  (0.1) | 3.11  (2.06-4.69) | 3.6x10^-05^ |
|  | H03AA01 | Levothyroxine sodium | 66 (12.4) | 237 (4.5) | 373/66 (5.6) | 1,416/237  (6.0) | 0.95  (0.74-1.20) | 6.5x10^-1^ | 373/533  (0.70) | 1,416/5,255  (0.3) | 2.60  (1.81-3.73) | 1.4x10^-04^ |
|  | J01MA12 | Levofloxacin | 79 (14.8) | 509(9.7) | 161/79 (2.0) | 882/509 (1.7) | 1.18  (0.98-1.41) | 8.2x10^-2^ | 161/533 (0.3) | 882/5,255  (0.2) | 1.80  (1.36-2.38) | 2.0x10^-02^ |
| **Specialist visits or laboratory/**  **diagnostic tests** | 90.15.2 | Corticotropin level measurement | 22 (4.1) | 3 (0.1) | 36/22 (1.6) | 3/3 (1.0) | 1.64  (0.21-12.83) | 6.4x10^-1^ | 36/533 (0.1) | 3/5,255  (0.0) | 118.31  (30.16-464.11) | 6.9x10^-09^ |
|  | 92.29.7 | Customized shielding | 5 (0.9) | 4 (0.1) | 22/5 (4.4) | 5/4 (1.3) | 3.52  (0.29-42.28) | 3.5x10^-1^ | 22/533 (0.0) | 5/5,255  (0.0) | 43.38  (9.33-201.80) | 1.3x10^-03^ |
|  | 90.15.3 | Cortisol level measurement | 36 (6.8) | 15 (0.3) | 67/36 (1.9) | 18/15 (1.2) | 1.55  (0.50-4.82) | 4.5x10^-1^ | 67/533 (0.1) | 18/5,255  (0.0) | 36.70  (17.41-77.37) | 3.3x10^-18^ |
|  | 91.48.5 | Arterial blood sampling | 3 (0.6) | 8 (0.2) | 20/3 (6.7) | 8/8 (1.0) | 6.67  (1.74-25.50) | 2.2x10^-2^ | 20/533 (0.0) | 8/5,255  (0.0) | 24.65  (7.19-84.49) | 2.9x10^-04^ |
|  | 92.18.6 | Total body tomography | 17 (3.2) | 12 (0.2) | 37/17 (2.2) | 16/12 (1.3) | 1.63  (0.88-3.01) | 1.3x10^-1^ | 37/533 (0.1) | 16/5,255  (0.0) | 22.80  (10.36-50.16) | 7.4x10^-12^ |
|  | 90.74.5 | Reticulocyte count | 4 (0.8) | 5 (0.1) | 13/4 (3.2) | 6/5 (1.2) | 2.71  (0.54-13.53) | 2.6x10^-1^ | 13/533 (0.0) | 6/5,255  (0.0) | 21.36  (5.56-82.11) | 6.9x10^-03^ |
|  | 92.29 | Other radiotherapeutic procedures | 7 (1.3) | 6 (0.1) | 10/7 (1.4) | 6/6 (1.0) | 1.43  (0.87-2.34) | 1.8x10^-1^ | 10/533 (0.0) | 6/5,255  (0.0) | 16.43  (5.76-46.85) | 1.4x10^-04^ |
|  | 92.40.2 | Intensity-modulated radiation therapy | 7 (1.3) | 5 (0.1) | 8/7 (1.1) | 5/5 (1.0) | 1.14  (0.84-1.55) | 4.1x10^-1^ | 8/533 (0.0) | 5/5,255  (0.0) | 15.77  (5.10-48.79) | 1.4x10^-03^ |
|  | 92.18.5 | Total body scintigraphy with immunological or receptor tracers | 7 (1.3) | 5 (0.1) | 8/7 (1.1) | 5/5 (1.0) | 1.14  (0.84-1.55) | 4.1x10^-1^ | 8/533 (0.0) | 5/5,255  (0.0) | 15.77  (5.10-48.79) | 1.4x10^-03^ |
|  | 88.91.2 | NMR imaging of brain and encephalic trunk | 35 (6.6) | 27 (0.5) | 45/35 (1.3) | 30/27(1.1) | 1.16  (0.90-1.49) | 2.6x10^-1^ | 45/533 (0.1) | 30/5,255  (0.0) | 14.79  (8.75-25.00) | 1.1x10^-20^ |
|  | 88.01.2 | CT of upper abdomen with or without contrast agents | 8 (1.5) | 10 (0.2) | 15/8(1.9) | 10/10 (1.0) | 1.88  (0.93-3.77) | 9.7x10^-2^ | 15/533 (0.0) | 10/5,255  (0.0) | 14.79  (6.12-35.75) | 1.9x10^-06^ |
|  | 88.01.4 | CT of lower abdomen with or without contrast agents | 7 (1.3) | 10 (0.2) | 14/7 (2.0) | 10/10 (1.0) | 2.00  (1.00-4.01) | 6.9x10^-2^ | 14/533 (0.0) | 10/5,255  (0.0) | 13.80  (5.61-33.99) | 9.7x10^-06^ |
|  | 90.41.3 | Testosterone level measurement | 30 (5.6) | 25 (0.5) | 44/30 (1.5) | 32/25 (1.3) | 1.15  (0.83-1.57) | 4.0x10^-1^ | 44/533 (0.1) | 32/5,255  (0.0) | 13.56  (7.62-24.11) | 7.5x10^-16^ |
|  | 87.03.8 | CT of neck with or without contrast agents | 8 (1.5) | 9 (0.2) | 11/8 (1.4) | 9/9 (1.0) | 1.38  (0.94-2.01) | 1.2x10^-1^ | 11/533 (0.0) | 9/5,255  (0.0) | 12.05  (4.86-29.90) | 6.7x10^-05^ |
|  | 87.03.1 | CT of head with or without contrast agents | 17 (3.2) | 17 (0.3) | 29/17 (1.7) | 26/17 (1.5) | 1.12  (0.56-2.22) | 7.6x10^-1^ | 29/533 (0.1) | 26/5,255  (0.0) | 11.00  (3.96-30.53) | 3.5x10^-03^ |
|  | 90.32.3 | Luteotropin level measurement | 29 (5.4) | 27 (0.5) | 32/29 (1.1) | 32/27 (1.2) | 0.93  (0.77-1.12) | 4.5x10^-1^ | 32/533 (0.1) | 32/5,255  (0.0) | 9.86  (5.64-17.22) | 8.8x10^-13^ |
|  | 92.29.8 | Customized immobilization system | 7 (1.3) | 8 (0.2) | 8/7 (1.1) | 8/8 (1.0) | 1.14  (0.90-1.45) | 2.9x10^-1^ | 8/533 (0.0) | 8/5,255  (0.0) | 9.86  (3.66-26.53) | 4.9x10^-03^ |
|  | 92.29.2 | Identification of clinical target volume and simulation (with CT) | 7 (1.3) | 8 (0.2) | 8/7 (1.1) | 9/8 (1.1) | 1.02  (0.73-1.41) | 9.3x10^-1^ | 8/533 (0.0) | 9/5,255  (0.0) | 8.76  (3.06-25.12) | 4.4x10^-02^ |
|  | 88.91.1 | NMR imaging of brain and encephalic trunk with and without contrast agents | 15 (2.8) | 18 (0.3) | 15/15 (1.0) | 19/18 (1.1) | 0.95  (0.84-1.06) | 3.6x10^-1^ | 15/533 (0.0) | 19/5,255  (0.0) | 7.78  (3.84-15.78) | 1.1x10^-05^ |
|  | 90.28.4 | Immunoglobulins: kappa and lambda chains | 21 (3.9) | 32 (0.6) | 28/21 (1.3) | 36/32 (1.1) | 1.19  (0.93-1.52) | 1.8x10^-1^ | 28/533 (0.1) | 36/5,255  (0.0) | 7.67  (4.40-13.36) | 5.8x10^-10^ |
|  | 90.69.4 | Immunoglobulins IgA, IgG or IgM | 8 (1.5) | 22 (0.4) | 28/8 (3.5) | 36/22 (1.6) | 2.14  (1.10-4.14) | 3.2x10^-2^ | 28/533 (0.1) | 36/5,255  (0.0) | 7.67  (3.24-18.16) | 3.0x10^-03^ |
|  | 90.61.4 | d-dimer level measurement | 9 (1.7) | 16 (0.3) | 13/9 (1.4) | 17/16 (1.1) | 1.36  (0.96-1.92) | 9.3x10^-2^ | 13/533 (0.0) | 17/5,255  (0.0) | 7.54  (3.43-16.56) | 4.1x10^-04^ |
|  | 90.23.3 | Follitropin level measurement | 27 (5.1) | 34 (0.6) | 31/27 (1.1) | 42/34 (1.2) | 0.93  (0.77-1.12) | 4.5x10^-1^ | 31/533 (0.1) | 42/5,255  (0.0) | 7.28  (4.23-12.51) | 6.5x10^-10^ |
|  | 90.55.1 | Carbohydrate antigen 125 level measurement | 20 (3.8) | 55 (1.0) | 59/20 (2.9) | 86/55 (1.6) | 1.89  (1.21-2.95) | 6.8x10^-3^ | 59/533 (0.1) | 86/5,255  (0.0) | 6.76  (3.92-11.66) | 5.4x10^-09^ |
|  | 87.41.1 | CT of chest with or without contrast agents | 34 (6.4) | 50 (1.0) | 56/34 (1.6) | 83/50 (1.7) | 0.99  (0.70-1.41) | 9.7x10^-1^ | 56/533 (0.1) | 83/5,255  (0.0) | 6.65  (3.77-11.73) | 5.1x10^-08^ |
|  | 87.41 | CT of chest | 13 (2.4) | 27 (0.5) | 18/13 (1.4) | 31/27 (1.2) | 1.21  (0.86-1.69) | 2.8x10^-1^ | 18/533 (0.0) | 31/5,255  (0.0) | 5.72  (2.90-11.32) | 4.4x10^-04^ |
|  | 88.01.6 | CT of abdomen with or without contrast agents | 34 (6.4) | 45 (0.9) | 48/34 (1.4) | 83/45 (1.8) | 0.77  (0.54-1.09) | 1.4x10^-1^ | 48/533 (0.1) | 83/5,255  (0.0) | 5.70  (3.07-10.58) | 2.9x10^-05^ |
|  | 90.35.5 | Parathyroid hormone | 30 (5.6) | 61 (1.2) | 54/30 (1.8) | 95/61 (1.6) | 1.16  (0.83-1.61) | 3.9x10^-1^ | 54/533 (0.1) | 95/5,255  (0.0) | 5.60  (3.35-9.38) | 4.6x10^-08^ |
|  | 90.55.3 | Carbohydrate antigen 19.9 | 31 (5.8) | 71 (1.4) | 66/31 (2.1) | 120/71 (1.7) | 1.26  (0.94-1.69) | 1.3x10^-1^ | 66/533 (0.1) | 120/5,255  (0.0) | 5.42  (3.37-8.71) | 2.6x10^-09^ |
|  | 90.24.5 | Phosphorus level measurement | 32 (6.0) | 94 (1.8) | 103/32 (3.2) | 214/94 (2.3) | 1.41  (0.94-2.12) | 9.5x10^-2^ | 103/533 (0.2) | 214/5,255  (0.0) | 4.75  (2.90-7.77) | 5.5x10^-07^ |
|  | 90.30.2 | Lipase level measurement | 29 (5.4) | 93 (1.8) | 55/29 (1.9) | 124/93 (1.3) | 1.42  (1.00-2.01) | 4.9x10^-2^ | 55/533 (0.1) | 124/5,255  (0.0) | 4.37  (2.73-7.00) | 7.2x10^-07^ |
|  | 88.71.4 | Ultrasound diagnostics of the head and neck | 35 (6.6) | 69 (1.3) | 40/35 (1.1) | 91/69 (1.3) | 0.87  (0.73-1.03) | 1.0x10^-1^ | 40/533 (0.1) | 91/5,255  (0.0) | 4.33  (2.74-6.85) | 3.1x10^-07^ |
|  | 90.38.5 | Proteins level measurement | 30 (5.6) | 99 (1.9) | 72/30 (2.4) | 170/99 (1.7) | 1.40  (0.93-2.11) | 1.1x10^-1^ | 72/533 (0.1) | 170/5,255  (0.0) | 4.18  (2.55-6.85) | 1.2x10^-05^ |
|  | 90.21.4 | Faecal occult blood | 16 (3.0) | 41 (0.8) | 21/16 (1.3) | 50/41 (1.2) | 1.08  (0.84-1.38) | 5.6x10^-1^ | 21/533 (0.1) | 50/5,255  (0.0) | 4.14  (2.26-7.59) | 3.6x10^-03^ |
|  | 90.11.4 | Total calcium level measurement | 90 (16.9) | 338 (6.4) | 277/90 (3.1) | 681/338 (2.0) | 1.53  (1.25-1.87) | 5.0x10^-5^ | 277/533 (0.5) | 681/5,255  (0.1) | 4.01  (3.07-5.24) | 4.4x10^-21^ |
|  | 87.03 | CT of head | 20 (3.8) | 51 (1.0) | 25/20 (1.2) | 63/51 (1.2) | 1.01  (0.78-1.31) | 9.3x10^-1^ | 25/533 (0.1) | 63/5,255  (0.0) | 3.91  (2.19-6.99) | 3.4x10^-03^ |
|  | 90.14.4 | Pseudocholinesterase | 16 (3.0) | 55 (1.0) | 25/16 (1.6) | 67/55 (1.2) | 1.28  (0.98-1.68) | 7.7x10^-2^ | 25/533 (0.1) | 67/5,255  (0.0) | 3.68  (2.09-6.47) | 5.0x10^-03^ |
|  | 90.16.4 | Creatinine clearance | 35 (6.6) | 118 (2.2) | 67/35 (1.9) | 186/118 (1.6) | 1.21  (0.85-1.73) | 2.9x10^-1^ | 67/533 (0.1) | 186/5,255  (0.0) | 3.55  (2.28-5.53) | 1.8x10^-05^ |
|  | 90.06.4 | Alpha amylase level measurement | 35 (6.6) | 131 (2.5) | 65/35 (1.9) | 187/131 (1.4) | 1.30  (0.98-1.73) | 7.4x10^-2^ | 65/533 (0.1) | 187/5,255  (0.0) | 3.43  (2.24-5.24) | 1.1x10^-05^ |
|  | 90.56.3 | Carcino-Embryonic Antigen | 30 (5.6) | 109 (2.1) | 73/30 (2.4) | 212/109 (1.9) | 1.25  (0.95-1.66) | 1.2x10^-1^ | 73/533 (0.1) | 212/5,255  (0.0) | 3.39  (2.14-5.39) | 1.7x10^-04^ |
|  | 90.29.2 | Lactate dehydrogenase level measurement | 59 (11.1) | 237 (4.5) | 135/59 (2.3) | 396/237 (1.7) | 1.37  (1.12-1.68) | 2.9x10^-3^ | 135/533  (0.3) | 396/5,255  (0.1) | 3.36  (2.45-4.62) | 7.0x10^-11^ |
|  | 90.10.4 | Total bilirubin level measurement | 43 (8.1) | 179 (3.4) | 85/43 (2.0) | 251/179 (1.4) | 1.41  (1.16-1.72) | 7.9x10^-4^ | 85/533 (0.2) | 251/5,255  (0.1) | 3.34  (2.37-4.70) | 4.9x10^-09^ |
|  | 90.13.3 | Chloride level measurement | 38 (7.1) | 133 (2.5) | 80/38 (2.1) | 249/133 (1.9) | 1.12 (0.84-1.51) | 4.4x10^-1^ | 80/533 (0.2) | 249/5,255 (0.1) | 3.17 (2.02-4.97) | 4.2x10^-04^ |
|  | 90.54.4 | Antithyroglobulin antibodies level measurement | 42 (7.9) | 144 (2.7) | 60/42 (1.4) | 193/144 (1.3) | 1.07  (0.85-1.34) | 5.9x10^-1^ | 60/533 (0.1) | 193/5,255  (0.0) | 3.07  (2.05-4.58) | 4.2x10^-05^ |
|  | 90.23.5 | Alkaline phosphatase level measurement | 74 (13.9) | 326 (6.2) | 182/74 (2.5) | 595/326 (1.8) | 1.35  (1.12-1.62) | 1.7x10^-3^ | 182/533  (0.3) | 595/5,255  (0.1) | 3.02  (2.27-4.00) | 1.6x10^-11^ |
|  | 90.10.5 | Total and fractional bilirubin level measurement | 98 (18.4) | 474 (9.0) | 252/98 (2.6) | 853/474 (1.8) | 1.43  (1.21-1.68) | 2.0x10^-5^ | 252/533  (0.5) | 853/5,255  (0.2) | 2.91  (2.29-3.70) | 2.9x10^-15^ |
|  | 90.22.3 | Ferritin level measurement | 64 (12.0) | 299 (5.7) | 158/64 (2.5) | 537/299 (1.8) | 1.37  (1.09-1.73) | 6.6x10^-3^ | 158/533 (0.3) | 537/5,255  (0.1) | 2.90  (2.10-4.00) | 7.5x10^-08^ |
|  | 88.76.1 | Full abdomen ultrasound | 45 (8.4) | 205 (3.9) | 72/45 (1.6) | 251/205 (1.2) | 1.31  (1.10-1.55) | 2.2x10^-3^ | 72/533 (0.1) | 251/5,255  (0.1) | 2.83  (2.03-3.94) | 6.4x10^-07^ |
|  | 90.40.4 | Sodium level measurement | 129 (24.2) | 744 (14.2) | 425/129  (3.3) | 1,489/744  (2.0) | 1.65  (1.43-1.89) | 3.1x10^-12^ | 425/533  (0.8) | 1489/5,255  (0.3) | 2.81  (2.31-3.43) | 7.7x10^-22^ |
|  | 90.51.4 | Antimicrosomal antibodies or anti thyroid peroxidase antibodies | 37 (6.9) | 135 (2.6) | 46/37 (1.2) | 165/135 (1.2) | 1.02  (0.86-1.20) | 8.4x10^-1^ | 46/533 (0.1) | 165/5,255  (0.0) | 2.75  (1.86-4.06) | 3.0x10^-04^ |
|  | 90.37.4 | Potassium level measurement | 132 (24.8) | 801 (15.2) | 451/132 (3.4) | 1,626/801 (2.0) | 1.68  (1.47-1.93) | 1.4x10^-13^ | 451/533 (0.9) | 1,626/5,255  (0.3) | 2.73  (2.26-3.32) | 1.7x10^-21^ |
|  | 90.44.1 | Urea level measurement | 178 (33.4) | 1,091 (20.8) | 592/178  (3.3) | 2,190/1,091  (2.0) | 1.66  (1.46-1.88) | 3.9x10^-15^ | 592/533  (1.1) | 2,190/5,255  (0.4) | 2.67  (2.26-3.15) | 2.0x10^-27^ |
|  | 90.38.4 | Protein electrophoresis | 117  (22.0) | 549 (10.4) | 249/117  (2.1) | 924/549  (1.7) | 1.26  (1.10-1.45) | 9.6x10^-4^ | 249/533  (0.5) | 924/5,255  (0.2) | 2.66  (2.13-3.31) | 3.4x10^-15^ |
|  | 90.94.2 | Urine culture (Addis count) | 37 (6.9) | 174 (3.3) | 74/37 (2.0) | 276/174  (1.6) | 1.26  (0.96-1.65) | 9.1x10^-2^ | 74/533  (0.1) | 276/5,255  (0.1) | 2.64  (1.74-4.01) | 4.1x10^-03^ |
|  | 90.22.5 | Iron level measurement | 112  (21.0) | 663 (12.6) | 328/112 (2.9) | 1,240/663  (1.9) | 1.57  (1.34-1.82) | 1.2x10^-8^ | 328/533  (0.6) | 1,240/5,255  (0.2) | 2.61  (2.10-3.24) | 3.6x10^-15^ |
|  | 90.56.5 | Prostate-Specific Antigen | 49 (9.2) | 303 (5.8) | 149/49 (3.0) | 562/303  (1.9) | 1.64  (1.33-2.02) | 4.4x10^-6^ | 149/533  (0.3) | 562/5,255  (0.1) | 2.61  (1.90-3.59) | 2.6x10^-06^ |
|  | 90.16.3 | Creatinin level measurement | 185 (34.7) | 1,106  (21.0) | 616/185  (3.3) | 2,350/1,106  (2.1) | 1.57  (1.38-1.77) | 2.0x10^-12^ | 616/533  (1.2) | 2,350/5,255  (0.5) | 2.58  (2.19-3.06) | 1.7x10^-25^ |
|  | 90.76.1 | Partial thromboplastin time | 56 (10.5) | 258  (4.9) | 111/56  (2.0) | 437/258  (1.7) | 1.17  (0.89-1.54) | 2.6x10^-1^ | 111/533  (0.2) | 437/5,255  (0.1) | 2.50  (1.71-3.67) | 2.2x10^-03^ |
|  | 90.42.3 | Free thyroxine level measurement | 132  (24.8) | 507  (9.6) | 238/132  (1.8) | 948/507  (1.9) | 0.96  (0.82-1.13) | 6.6x10^-1^ | 238/533  (0.5) | 948/5,255  (0.2) | 2.48  (1.94-3.16) | 4.3x10^-10^ |
|  | 90.42.1 | Thyroid stimulating hormone level measurement | 138  (25.9) | 545  (10.4) | 253/138  (1.8) | 1,034/545  (1.9) | 0.97  (0.82-1.13) | 6.8x10^-1^ | 253/533  (0.5) | 1,034/5,255  (0.2) | 2.41  (1.90-3.07) | 7.4x10^-10^ |
|  | 90.43.3 | Free triiodothyronine level measurement | 124  (23.3) | 493  (9.4) | 219/124  (1.8) | 911/493  (1.9) | 0.96  (0.81-1.13) | 6.0x10^-1^ | 219/533  (0.4) | 911/5,255  (0.2) | 2.37  (1.84-3.06) | 3.2x10^-08^ |
|  | 90.65.1 | Functional fibrinogen level measurement | 58 (10.9) | 352  (6.7) | 127/58  (2.2) | 547/352  (1.6) | 1.41  (1.16-1.71) | 6.4x10^-4^ | 127/533  (0.2) | 547/5,255  (0.1) | 2.29  (1.69-3.10) | 7.5x10^-05^ |
|  | 90.62.2 | Blood count | 228 (42.8) | 1,676  (31.9) | 910/228  (4.0) | 3,928/1,676  (2.3) | 1.70  (1.51-1.92) | 2.5x10^-18^ | 910/533  (1.7) | 3,928/5,255  (0.8) | 2.28  (1.97-2.65) | 7.5x10^-25^ |
|  | 89.01 | Anamnesis and examination - short | 121 (22.7) | 577  (11.0) | 332/121  (2.7) | 1,443/577  (2.5) | 1.10  (0.90-1.34) | 3.6x10^-1^ | 332/533  (0.6) | 1443/5,255  (0.3) | 2.27  (1.74-2.95) | 1.0x10^-06^ |
|  | 90.72.3 | C-reactive protein level measurement | 68 (12.8) | 363  (6.9) | 139/68  (2.0) | 605/363  (1.7) | 1.23  (1.01-1.49) | 3.9x10^-2^ | 139/533  (0.3) | 605/5,255  (0.1) | 2.27  (1.68-3.05) | 6.1x10^-05^ |
|  | 90.82.5 | Erythrocyte Sedimentation Rate | 84 (15.8) | 480  (9.1) | 198/84  (2.4) | 870/480  (1.8) | 1.30  (1.10-1.53) | 1.7x10^-3^ | 198/533  (0.4) | 870/5,255  (0.2) | 2.24  (1.74-2.89) | 4.3x10^-07^ |
|  | 90.25.5 | Gamma-glutamyl Transferase level measurement | 106 (19.9) | 629  (12.0) | 257/106  (2.4) | 1,158/629  (1.8) | 1.32  (1.14-1.52) | 1.9x10^-4^ | 257/533  (0.5) | 1,158/5,255  (0.2) | 2.19  (1.75-2.74) | 5.9x10^-09^ |
|  | 91.49.2 | Venous blood sampling | 260  (48.8) | 1875  (35.7) | 1,250/260  (4.8) | 5,669/1,875  (3.0) | 1.59  (1.41-1.79) | 1.5x10^-14^ | 1250/533  (2.4) | 5,669/5,255  (1.1) | 2.17  (1.88-2.51) | 9.4x10^-23^ |
|  | 90.28.1 | Glycated hemoglobin level measurement | 67 (12.6) | 364  (6.9) | 181/67  (2.7) | 823/364  (2.3) | 1.19  (0.99-1.44) | 5.9x10^-2^ | 181/533  (0.3) | 823/5,255  (0.2) | 2.17  (1.62-2.91) | 1.9x10^-04^ |
|  | 89.7 | General medical examination | 233  (43.7) | 1597  (30.4) | 884/233  (3.8) | 4,056/1,597  (2.5) | 1.49  (1.32-1.68) | 7.9x10^-11^ | 884/533  (1.7) | 4,056/5,255  (0.8) | 2.15  (1.84-2.51) | 2.4x10^-19^ |
|  | 87.44.1 | Routine chest radiography | 44 (8.3) | 216  (4.1) | 57/44 (1.3) | 261/216  (1.2) | 1.07  (0.92-1.24) | 3.6x10^-1^ | 57/533  (0.1) | 261/5,255  (0.1) | 2.15  (1.53-3.04) | 9.9x10^-03^ |
|  | 90.43.5 | Urate level measurement | 122 (22.9) | 808  (15.4) | 331/122  (2.7) | 1,581/808  (2.0) | 1.39  (1.21-1.59) | 2.9x10^-6^ | 331/533  (0.6) | 1,581/5,255  (0.3) | 2.06  (1.69-2.53) | 2.2x10^-09^ |
|  | 90.27.1 | Glucose level measurement | 219  (41.1) | 1,558  (29.6) | 692/219  (3.2) | 3,343/1,558  (2.2) | 1.47  (1.33-1.63) | 5.3x10^-13^ | 692/533  (1.3) | 3,343/5,255  (0.6) | 2.04  (1.77-2.36) | 2.4x10^-19^ |
|  | 90.09.2 | Aspartate aminotransferase level measurement | 199  (37.3) | 1,417  (27.0) | 594/199  (3.0) | 2,905/1,417  (2.1) | 1.46  (1.30-1.63) | 5.6x10^-11^ | 594/533  (1.1) | 2,905/5,255  (0.6) | 2.02  (1.73-2.35) | 5.0x10^-16^ |
|  | 90.04.5 | Alanine aminotransferase level measurement | 195  (36.6) | 1,415  (26.9) | 586/195  (3.0) | 2,904/1,415  (2.1) | 1.46  (1.31-1.64) | 4.1x10^-11^ | 586/533  (1.1) | 2,904/5,255  (0.6) | 1.99  (1.70-2.32) | 4.3x10^-15^ |
|  | 90.14.3 | Total cholesterol level measurement | 183  (34.3) | 1,414  (26.9) | 457/183  (2.5) | 2,831/1,414  (2.0) | 1.25  (1.13-1.38) | 2.6x10^-5^ | 457/533  (0.9) | 2,831/5,255  (0.5) | 1.59  (1.36-1.86) | 6.1x10^-06^ |
|  | 90.44.3 | Complete urine culture | 182  (34.1) | 1,458  (27.7) | 473/182  (2.6) | 3,005/1,458  (2.1) | 1.26  (1.13-1.40) | 2.2x10^-5^ | 473/533  (0.9) | 3,005/5,255  (0.6) | 1.55  (1.32-1.82) | 5.4x10^-05^ |
|  | 90.14.1 | HDL cholesterol level measurement | 147  (27.6) | 1,105  (21.0) | 335/147  (2.3) | 2,170/1,105  (2.0) | 1.16  (1.04-1.30) | 1.0x10^-2^ | 335/533  (0.6) | 2,170/5,255  (0.4) | 1.52  (1.27-1.83) | 5.5x10^-03^ |
|  | 90.43.2 | Triglycerides level measurement | 171  (32.1) | 1,367  (26.0) | 404/171  (2.4) | 2,677/1,367  (2.0) | 1.21  (1.08-1.34) | 5.4x10^-14^ | 404/533  (0.8) | 2,677/5,255  (0.5) | 1.49  (1.26-1.75) | 1.9x10^-03^ |

**Abbreviations:** N**_subj_** = Number of subjects; N**_reg_**= Total number of (code) registrations; ATC = anatomical therapeutic chemical classification; CT = computed tomography; NMR = nuclear magnetic resonance; 95% CI = 95% confidence interval.

P-values are referred to the “mean ratio” between the mean number of registrations per subject in cases and controls and were computed from univariable over-dispersed Poisson regressions and then corrected for multiple testing, following the Bonferroni method. Only statistically significant mean ratios among all subjects (i.e., adjusted p-values <0.05) are shown.

*This measure is calculated as the ratio of the total number of (code) registrations to the number of subjects with at least one registration;

°This measure is calculated as the ratio of the total number of (code) registrations to the total number of subjects;

^#^This measure is calculated as the ratio of the mean number of registrations per subject between cases and controls and indicates how many times the mean number of registrations per subject in cases is higher than in matched controls

**Supplementary Table 3.** Optimal values set for tuning parameters and thresholds for each predictive model and algorithm.

| **Cross-Validated multivariable conditional logistic regression with LASSO penality** | | |
| --- | --- | --- |
| **Model output** | **Optimal tuning parameters** | **Optimal threshold** |
| Binary classification | Penality (lambda)= 27.4 | 0.51 |
| **Cross-Validated multivariable unconditional logistic regression with LASSO penality** | | |
| **Model output** | **Optimal tuning parameters** | **Optimal threshold** |
| Individual probabilities | Penality (lambda)= 0.03 | 0.087 |
| **Case-weighted (IPW) Recursive PArtitioning and Regression Tree (RPART)** | | |
| **Model output** | **Optimal tuning parameters** | **Optimal threshold** |
| Individual probabilities | Node size= 42  cp = 0.002 | 0.48 |
| **Case-weighted (IPW) Random Forest** | | |
| **Model output** | **Optimal tuning parameters** | **Optimal threshold** |
| Individual probabilities | N. trees= 100,000  Node size=150 | 0.55 |
| **Case-weighted (IPW) Support Vector Machine** | | |
| **Model output** | **Optimal tuning parameters** | **Optimal threshold** |
| Individual probabilities | Radial kernel (C = 0.25; Gamma = 0.05) | 0.06 |

**Abbreviations:** cp = complexity parameter; C = cost parameter; IPW: Inverse Probability Weighting; LASSO = Least Absolute Shrinkage and Selection Operator

**Supplementary Document 2.** Diagnostic accuracy of machine-learning algorithms in presence of data misclassification

1 – Counts of true positive (TP), false positive (FP), false negative (FN), true negative (TN) and diagnostic accuracy measures of the validated coding algorithm (A) used to detect the presence of acromegaly (D):

|  | **Disease = Yes (D^+^)** | **Disease = No (D^-^)** |
| --- | --- | --- |
| **A = Positive (A^+^)** | 32 (TP) | 60 (FP) |
| **A = Negative (A^-^)** | 6 (FN) | 1,060,806 (TN) |

SE_A_ = TP/D^+^ = 32 / 88 = 84.21%

SP_A_ = TN/D^-^ = 1,060,806 / 1,060,866 = 99.99%

The following counts and 2 × 2 contingency table can be found in Table 3 of *Crisafulli, S., Fontana, A., L’Abbate, L. et al. Development and testing of diagnostic algorithms to identify patients with acromegaly in Southern Italian claims databases. Sci Rep 12, 15843 (2022).* [*https://doi.org/10.1038/s41598-022-20295-4*](https://doi.org/10.1038/s41598-022-20295-4). The diagnosis of disease (D) was ascertained by the registration in the electronic therapeutic plans database with at least one ICD-9 CM code for acromegaly (i.e. the established gold standard).

2 – Counts of true positive (TP), false positive (FP), false negative (FN), true negative (TN) and diagnostic accuracy measures of the meta-score algorithm (machine-learning, ML) used to detect the positivity of the validated algorithm (A) in the test set of the present study (N=1,739 subjects):

|  | **A = Positive (A^+^)** | **A = Negative (A^-^)** |
| --- | --- | --- |
| **ML = Positive (ML^+^)** | 64 (TP) | 316 (FP) |
| **ML = Negative (ML^-^)** | 96 (FN) | 1,263 (TN) |

SE_ML|A_ = TP/A^+^ = 64 / 160 = 40.00%

SP_ML|A_ = TN/A^-^ = 1,263 / 1,579 = 79.99%

Apparent prevalence (Pr) = A^+^ / N = 160 / 1,739 = 0.092 = 9.2 cases per 100 subjects

The validated coding algorithm represents the reference standard test.

3 – Calculation of the diagnostic accuracy measures of the meta-score algorithm (machine-learning, ML) that would have been found if the subjects in the test set had been classified with the established gold standard:

$$\text{SE}_{\text{ML}}\text{ = }\frac{Pr\cdot{SE}_{ML|A}\cdot{SP}_{A}-(1-Pr)(1-{SP}_{ML|A})(1-{SP}_{A})}{Pr+{SP}_{A}-1}\text{ = }\frac{\text{0.03679}}{\text{0.09195}}\text{ = 40.01\%}$$

$$\text{SP}_{\text{ML}}\text{ = }\frac{Pr\cdot{(1-SE}_{ML|A})+ {SE}_{A}\cdot[Pr\cdot\left( {SE}_{ML|A}-1 \right)-(1-Pr)\cdot{SP}_{ML|A}]}{Pr-{SE}_{A}}\text{ = }\frac{\text{-}\text{0.}\text{6029}}{\text{-0.7501}}\text{ = }\text{80}\text{.37\%}$$

4 – Calculation of the diagnostic accuracy measures of any machine-learning (ML) algorithm that would be found if subjects in the test set were classified by the established gold standard. To this end, knowing that A^+^ = 160 and that A^-^ = 1,579, the theoretical counts of TP, FP, FN and TN were computed assuming different percentages (from 0% to 100% by 25%) of TP among the A^+^ and TN among the A^-^. Both the sensitivity (SE) and specificity (SP) of the ML algorithm estimated using the reference standard test were denoted with the subscript “ML|A” while the (estimated) theoretical SE and SP that would have been found if the subjects had been classified with the established gold standard were denoted with the subscript “ML”. If the theoretical values were less than zero or greater than one, they were rounded to zero and one respectively. The absolute differences between actual and theoretical values define the estimated bias.

| **% TP** | **% TN** | **TP** | **FP** | **FN** | **TN** | **SE_ML\|A_** | **SP_ML\|A_** | **SE_ML_** | **SP_ML_** | **Bias SE** | **Bias SP** |
| --- | --- | --- | --- | --- | --- | --- | --- | --- | --- | --- | --- |
| 0% | 0% | 0 | 160 | 1579 | 0 | 0.00 | 0.00 | 0.0000 | 0.0000 | 0.0000 | 0.0000 |
|  | 25% | 0 | 160 | 1184.25 | 394.75 | 0.00 | 0.25 | 0.0000 | 0.2355 | 0.0000 | 0.0145 |
|  | 50% | 0 | 160 | 789.5 | 789.5 | 0.00 | 0.50 | 0.0000 | 0.4903 | 0.0000 | 0.0097 |
|  | 75% | 0 | 160 | 394.75 | 1184.25 | 0.00 | 0.75 | 0.0000 | 0.7452 | 0.0000 | 0.0048 |
|  | 100% | 0 | 160 | 0 | 1579 | 0.00 | 1.00 | 0.0000 | 1.0000 | 0.0000 | 0.0000 |
| 25% | 0% | 40 | 120 | 1579 | 0 | 0.25 | 0.00 | 0.2496 | 0.0000 | 0.0004 | 0.0000 |
|  | 25% | 40 | 120 | 1184.25 | 394.75 | 0.25 | 0.25 | 0.2497 | 0.2403 | 0.0003 | 0.0097 |
|  | 50% | 40 | 120 | 789.5 | 789.5 | 0.25 | 0.50 | 0.2499 | 0.4952 | 0.0001 | 0.0048 |
|  | 75% | 40 | 120 | 394.75 | 1184.25 | 0.25 | 0.75 | 0.2500 | 0.7500 | 0.0000 | 0.0000 |
|  | 100% | 40 | 120 | 0 | 1579 | 0.25 | 1.00 | 0.2501 | 1.0000 | 0.0001 | 0.0000 |
| 50% | 0% | 80 | 80 | 1579 | 0 | 0.50 | 0.00 | 0.4997 | 0.0000 | 0.0003 | 0.0000 |
|  | 25% | 80 | 80 | 1184.25 | 394.75 | 0.50 | 0.25 | 0.4999 | 0.2452 | 0.0001 | 0.0048 |
|  | 50% | 80 | 80 | 789.5 | 789.5 | 0.50 | 0.50 | 0.5000 | 0.5000 | 0.0000 | 0.0000 |
|  | 75% | 80 | 80 | 394.75 | 1184.25 | 0.50 | 0.75 | 0.5001 | 0.7548 | 0.0001 | 0.0048 |
|  | 100% | 80 | 80 | 0 | 1579 | 0.50 | 1.00 | 0.5003 | 1.0000 | 0.0003 | 0.0000 |
| 75% | 0% | 120 | 40 | 1579 | 0 | 0.75 | 0.00 | 0.7499 | 0.0000 | 0.0001 | 0.0000 |
|  | 25% | 120 | 40 | 1184.25 | 394.75 | 0.75 | 0.25 | 0.7500 | 0.2500 | 0.0000 | 0.0000 |
|  | 50% | 120 | 40 | 789.5 | 789.5 | 0.75 | 0.50 | 0.7501 | 0.5048 | 0.0001 | 0.0048 |
|  | 75% | 120 | 40 | 394.75 | 1184.25 | 0.75 | 0.75 | 0.7503 | 0.7597 | 0.0003 | 0.0097 |
|  | 100% | 120 | 40 | 0 | 1579 | 0.75 | 1.00 | 0.7504 | 1.0000 | 0.0004 | 0.0000 |
| 100% | 0% | 160 | 0 | 1579 | 0 | 1.00 | 0.00 | 1.0000 | 0.0000 | 0.0000 | 0.0000 |
|  | 25% | 160 | 0 | 1184.25 | 394.75 | 1.00 | 0.25 | 1.0000 | 0.2548 | 0.0000 | 0.0048 |
|  | 50% | 160 | 0 | 789.5 | 789.5 | 1.00 | 0.50 | 1.0000 | 0.5097 | 0.0000 | 0.0097 |
|  | 75% | 160 | 0 | 394.75 | 1184.25 | 1.00 | 0.75 | 1.0000 | 0.7645 | 0.0000 | 0.0145 |
|  | 100% | 160 | 0 | 0 | 1579 | 1.00 | 1.00 | 1.0000 | 1.0000 | 0.0000 | 0.0000 |

**Mean bias:** SE=0.0001, SP=0.0039

**Supplementary Document 3.** Structure of the R code used to perform machine-learning algorithms. Note: This document does not include all the R codes, but only summarizes all the steps taken to apply the main functions required to build the algorithms

**# Splitting data in training and test set**

1. Extraction of 70% of the random sample strata from the matched dataset (i.e. 373 of 533 matching strata “id_strata”) and creation of training and test datasets. Each stratum is numbered from 1 to 533 and consists of 1 case and approximately 10 controls.

set.seed(1234)

sel_strata_train<-sample(1:533,373)

matched_data <- matched_data %>%

mutate(training=case_when(id_strata %in% sel_strata_train ~ 1, TRUE ~ 0)) %>%

arrange(id_strata, acromegaly)

train_db <- matched_data %>% filter(training == 1)

test_db <- matched_data %>% filter(training == 0)

**# Multivariable conditional logistic regression using LASSO penalty**

1. Fitting a multivariable conditional logistic regression model with LASSO penalty using the *clogitL1* function (from *clogitL1* package)

x <- data.matrix(train_db[,-c(1:6, dim(train_db)[2])]) #removing useless features and outcome

y <- train_db$acromegaly

#counting the number of observations within each stratum

nobs <- train_db %>% group_by(id_strata) %>% count()

#creation of a new “strata_id” ordered from 1 to 373

#as a result of random sampling, the old id_strata was shuffled)

strata <- rep(1:373, as.vector(nobs$n))

lasso_train <- clogitL1(x = x,

y = y,

strata = strata,

numLambda = 100,

minLambdaRatio=0.005,

switch = 0, #Note: switch = 90 was also evaluated!

alpha = 1) #when alpha = 1 the LASSO penalty is used

1a) Setting the number of different values of the regularization parameter (numLambda = 100), which means that the algorithm explored a maximum of 100 different values of the regularization parameter (from largest to smallest lambdas)

1b) Setting the ratio of the smallest value of regularization parameter to the largest one (minLambdaRatio=0.005), meaning that the smallest lambda was 0.005 times the largest one

1c) Setting the value of the switch parameter. If switch = 0 lambda values jump from largest to the smallest in logarithmic steps, whereas if switch = 90 the lambda values jump in linear steps for the first 90 numLambdas and in logarithmic steps for the last 10 numLambdas

1. Performing 10-fold cross-validation (CV) using the *cv.clogitL1* function

set.seed(1234)

cv_result <- cv.clogitL1(lasso_train, numFolds=10)

1. Extraction of the optimal lambda value that produced the minimum CV error (and the one that lies within one standard error of the optimal)

min_lambda <- exp(cv_result$minCV_lambda)

min1SE_lambda <- exp(cv_result$minCV1se_lambda)

1. *Extraction of the regression coefficients corresponding to min_lambda (the CV error was lower than the one achieved by min1SE_lambda)*
2. *Creation of the linear predictor (LP) variable as a linear combination of the selected features weighted by their regression coefficients in training dataset and test set*
3. Computation of the AUC of the LP in training and test set using roc function (package: pROC):

lasso_auc_train <- roc(train_db$acromegaly, train_db$lp, ci=TRUE)

lasso_auc_test <- roc(test_db$acromegaly, test_db$lp, ci=TRUE)

1. Defining the threshold of the LP which maximize the Youden Index and the subsequent computation of sensitivity, specificity, PPV, NPV, F-score in training and test set

best_lpcutoff<-as.numeric(coords(lasso_auc_train, "best", ret="threshold"))

pars_bestcut_train<-coords(lasso_auc_train, best_lpcutoff, ret=c("threshold", "specificity", "sensitivity", "tn", "tp", "fn", "fp"))

pars_bestcut_test<-coords(lasso_auc_test, best_lpcutoff, ret=c("threshold", "specificity", "sensitivity", "tn", "tp", "fn", "fp"))

#computing PPV, NPV, Youden Index, F-score

pars_bestcut_train <- pars_bestcut_train %>%

mutate(PPV=tp/(tp+fp),

NPV=tn/(tn+fn),

Youden=sensitivity + specificity - 1,

F_score= 2*((PPV*sensitivity)/(PPV+sensitivity)))

pars_bestcut_test <- pars_bestcut_test %>%

mutate(PPV=tp/(tp+fp),

NPV=tn/(tn+fn),

Youden=sensitivity + specificity - 1,

F_score= 2*((PPV*sensitivity)/(PPV+sensitivity)))

**# Multivariable unconditional logistic regression using LASSO penalty**

1. Fitting 10-fold CV multivariable unconditional logistic regression models with LASSO penalty using the *cv.glmnet* function (from *glmnet* package)

uncond_cv_result <- cv.glmnet(x = x,

y = y,

alpha = 1,

family = "binomial",

type.measure = "deviance",

nfolds = 10)

1a) x and y (i.e. matrix of features and outcome of the training dataset respectively) are defined previously

1b) Setting both family = "binomial" and type.measure = "deviance" refers to binomial deviance

1. Extraction of the optimal lambda value that produced the minimum CV error (and the one that lies within one standard error of the optimal)

uncond_min_lambda <- uncond_cv_result$lambda.min

uncond_min1SE_lambda <- uncond_cv_result$lambda.1se

1. Using the *predict.glmnet* function to define the variable with estimated individual probabilities corresponding to min1SE_lambda (the CV error was lower than the one achieved by min_lambda)

train_db <- train_db %>%

mutate(prob_ulasso = predict(uncond_cv_result,

newx = x,

s = uncond_min1SE_lambda,

type="response"))

test_db <- test_db %>%

mutate(prob_ulasso = predict(uncond_cv_result,

newx = as.matrix(test_db[,-c(1:6)]),

s = uncond_min1SE_lambda,

type="response"))

1. Computation of the AUC of the estimated individual probabilities in training and test set

ulasso_auc_train <- roc(train_db$acromegaly, train_db$prob_ulasso, ci=TRUE)

ulasso_auc_test <- roc(test_db$acromegaly, test_db$prob_ulasso, ci=TRUE)

1. Defining the threshold of the individual probabilities which maximize the Youden Index and the subsequent computation of sensitivity, specificity, PPV, NPV, F-score and ICI in training and test set

best_lpcutoff<-as.numeric(coords(ulasso_auc_train, "best", ret="threshold"))

pars_bestcut_train<-coords(ulasso_auc_train, best_lpcutoff, ret=c("threshold", "specificity", "sensitivity", "tn", "tp", "fn", "fp"))

pars_bestcut_test<-coords(ulasso_auc_test, best_lpcutoff, ret=c("threshold", "specificity", "sensitivity", "tn", "tp", "fn", "fp"))

#computing PPV, NPV, Youden Index, F-score

pars_bestcut_train <- pars_bestcut_train %>%

mutate(PPV=tp/(tp+fp),

NPV=tn/(tn+fn),

Youden=sensitivity + specificity - 1,

F_score= 2*((PPV*sensitivity)/(PPV+sensitivity)))

pars_bestcut_test <- pars_bestcut_test %>%

mutate(PPV=tp/(tp+fp),

NPV=tn/(tn+fn),

Youden=sensitivity + specificity - 1,

F_score= 2*((PPV*sensitivity)/(PPV+sensitivity)))

#ICI in training set

gam.calibrate_train <- gam(as.numeric(train_db$acromegaly)-1 ~ s(train_db$prob_ulasso, bs = 'cs'), method = 'REML')

p.calibrate_train <- predict(gam.calibrate_train)

ici_train<-mean(abs(p.calibrate_train - train_db$prob_ulasso))

#ICI in test set

gam.calibrate_test <- gam(as.numeric(test_db$acromegaly)-1 ~ s(test_db$prob_ulasso, bs = 'cs'), method = 'REML')

p.calibrate_test <- predict(gam.calibrate_test)

ici_test<-mean(abs(p.calibrate_test - test_db$prob_ulasso))

**# Recursive Partitioning and Regression Trees (RPART)**

1. Fitting 10-fold CV classification trees in the training dataset using the *rpart* function (from *rpart* package)

tree_train<-rpart(formula=acromegaly~.,

data=train_db,

weights=ipw_weights,

control=rpart.control(minsplit=50,

xval=10,

cp=0))

1a) Setting individual probability weights (ipw) for each subject as follows:

ipw_weights<-ifelse(train_db$acromegaly==1,

dim(train_db)[1]/sum(train_db$acromegaly),

dim(train_db)[1]/(dim(train_db)[1]-sum(train_db$acromegaly)))

1b) Building each CV tree setting with at least 50 ipw observations per node of the tree (minsplit=50). Setting the complexity parameter to zero (cp=0), several trees were built by varying the complexity parameter from 0, which results in the largest (unpruned) tree, to infinity, which results in the most pruned tree. At each tree the 10-fold CV classification error was evaluated.

1c) Pruning the tree step (using the *prune* function) the complexity parameter was set as the one that minimized the 10-fold CV classification error:

set.seed(1234)

pruned_tree_train<-prune(tree_train, cp=0.002)

1. Using the *predict.rpart* function to define the variable with estimated individual probabilities

train_db <- train_db %>%

mutate(prob_rpart = predict(pruned_tree_train, train_db, response="prob")[,2])

test_db <- test_db %>%

mutate(prob_rpart = predict(pruned_tree_train, test_db, response="prob")[,2])

1. Computation of the AUC of the estimated individual probabilities in training and test set

rpart_auc_train <- roc(train_db$acromegaly, train_db$prob_rpart, ci=TRUE)

rpart_auc_test <- roc(test_db$acromegaly, test_db$prob_rpart, ci=TRUE)

1. Defining the threshold of the individual probabilities which maximize the Youden Index and the subsequent computation of sensitivity, specificity, PPV, NPV, F-score and ICI in training and test set

best_lpcutoff<-as.numeric(coords(rpart_auc_train, "best", ret="threshold"))

pars_bestcut_train<-coords(rpart_auc_train, best_lpcutoff, ret=c("threshold", "specificity", "sensitivity", "tn", "tp", "fn", "fp"))

pars_bestcut_test<-coords(rpart_auc_test, best_lpcutoff, ret=c("threshold", "specificity", "sensitivity", "tn", "tp", "fn", "fp"))

#computing PPV, NPV, Youden Index, F-score

pars_bestcut_train <- pars_bestcut_train %>%

mutate(PPV=tp/(tp+fp),

NPV=tn/(tn+fn),

Youden=sensitivity + specificity - 1,

F_score= 2*((PPV*sensitivity)/(PPV+sensitivity)))

pars_bestcut_test <- pars_bestcut_test %>%

mutate(PPV=tp/(tp+fp),

NPV=tn/(tn+fn),

Youden=sensitivity + specificity - 1,

F_score= 2*((PPV*sensitivity)/(PPV+sensitivity)))

#ICI in training set

gam.calibrate_train <- gam(as.numeric(train_db$acromegaly)-1 ~ s(train_db$prob_rpart, bs = 'cs'), method = 'REML')

p.calibrate_train <- predict(gam.calibrate_train)

ici_train<-mean(abs(p.calibrate_train - train_db$prob_rpart))

#ICI in test set

gam.calibrate_test <- gam(as.numeric(test_db$acromegaly)-1 ~ s(test_db$prob_rpart, bs = 'cs'), method = 'REML')

p.calibrate_test <- predict(gam.calibrate_test)

ici_test<-mean(abs(p.calibrate_test - test_db$prob_rpart))

**# Random Forest**

1. Fitting random forest (RF) algorithms, using the *ranger* function (from *ranger* package), according to different minimum node size. Each RF has the following structure:

rf <- ranger(formula= acromegaly ~.,

data = train_db,

num.trees = 100000,

importance = "permutation"

scale.permutation.importance = FALSE,

local.importance = FALSE,

write.forest = TRUE,

probability = TRUE,

min.node.size = minnodesize,

max.depth = NULL,

replace = TRUE,

case.weights = ipw_weights,

splitrule = "gini",

regularization.factor = 1,

regularization.usedepth = FALSE,

oob.error = TRUE,

keep.inbag = FALSE,

save.memory = FALSE,

verbose = FALSE,

seed = 1234)

1a) The number of trees was fixed to 100,000 (num.trees = 100000)

1b) The following minimum node sizes (of each tree in the forest) were tried:

min.node.size = 50,150,300, 500.The highest AUC in the test set was achieved with size of 150.

1c) The permutation method was set to compute the variable importance (importance = "permutation")

1d) The algorithm was forced to return predicted probabilities (probability = TRUE)

1e) The ipw weights were assigned to observations (case.weights = ipw_weights)

1f) The Gini impurity criterion was set as splitting rule (splitrule = "gini") used to build the trees.

1g) No regularization was considered for the forest (regularization.factor = 1)

1h) Calculation of the out-of-bag prediction error (oob.error = TRUE)

1. The *predict.ranger* function was used to define the variable with estimated individual probabilities (the R code is omitted here because it is identical to the that reported for **RPART**)
2. Computation of the AUC of the estimated individual probabilities in training and test set (the R code is omitted here because it is identical to the that reported for **RPART**)
3. Defining the threshold of the individual probabilities which maximize the Youden Index and the subsequent computation of sensitivity, specificity, PPV, NPV, F-score and ICI in training and test set (the R code is omitted here because it is identical to the that reported for **RPART**)

**# Support Vector Machine**

1) Fitting 10-fold CV Support Vector Machine algorithm using the *train* function (from *caret* package). The CV was performed using the grid search for cost (C) and gamma parameters (tuning the hyperparameters):

svmGrid <- expand.grid(C = c(2^(1:4)),gamma = c(2^(-1:1))

ctrl <- trainControl(method="repeatedcv", number=10, repeats=3))

svmFit<-train(acromegaly ~.,

data=train_db,

method="svmRadial",

weights=ipw_weights,

trControl = ctrl,

tuneGrid = svmGrid)

1a) Regarding the cross-validation procedure, the number of folds is set to 10 (number=10), and the number of repeats is set to 3 (repeats=3)

1b) The radial basis kernel was used (method="svmRadial")

1c) The ipw weights were assigned to observations (weights=ipw_weights)

1. The *predict.train* function was used to define the variable with estimated individual probabilities, setting response="prob" (the R code is omitted here because it is identical to the that reported for **RPART**)
2. Computation of the AUC of the estimated individual probabilities in training and test set (the R code is omitted here because it is identical to the that reported for **RPART**)
3. Defining the threshold of the individual probabilities which maximize the Youden Index and the subsequent computation of sensitivity, specificity, PPV, NPV, F-score and ICI in training and test set (the R code is omitted here because it is identical to the that reported for **RPART**)
